# A Meta-Analysis of Atypical Sexuality, Psychopathy, and Recidivism Associated With Victim Age Polymorphism

**DOI:** 10.1177/10790632261415817

**Published:** 2026-01-24

**Authors:** Samantha K. Williams, Desiree L. Elchuk, Skye Stephens

**Affiliations:** 1 Department of Psychology, 3690 Saint Mary’s University, Halifax, NS, Canada

**Keywords:** victim age polymorphism, crossover offending, mixed offending, victim age

## Abstract

Victim age polymorphism describes a pattern of sexual offending in which individuals target victims from multiple distinct age categories (e.g., both child and adult victims). Research on victim age polymorphism and its association with risk-related domains — namely atypical sexuality and antisociality — and recidivism is mixed, potentially due to methodological differences across studies (e.g., how victim age is classified). This meta-analysis (*k* = 23, *N* = 12,333) examined associations between victim age polymorphism, the two main risk-related domains (atypical sexuality, antisociality), and recidivism. Meta-regression and the between-level *Q* statistic were used to examine various methodological differences that might contribute to disparate findings. Results indicated that victim age polymorphism was associated with multiple paraphilias, psychopathy, and recidivism. Moderator analyses were limited due to the small number of studies and did not consistently explain the variation in effect sizes. Overall, individuals who are polymorphic share more clinically relevant similarities to individuals who offend exclusively against adults than those who exclusively offend against children. These findings suggest that a greater focus on generalist criminogenic needs (e.g., antisociality) may be warranted in the management of individuals who are polymorphic.

## Introduction

Individuals who sexually offend are a heterogenous group that differ on a wide range of characteristics, including victim selection. One characteristic that is often used to distinguish individuals with sexual offenses is victim age (Aebi et al., 2011; Faniff & Kolko, 2011; Leroux et al., 2014). Conventionally, forensic researchers have categorized individuals with sexual offenses into those who sexually offend against children or those who sexually offend against adults; however, there is a group of individuals for whom victim selection is more variable (e.g., Brown et al., 2015; Cormier et al., 2020; Lussier et al., 2007). Victim age polymorphism describes a pattern of sexual offending in which individuals target victims from multiple distinct age categories (e.g., both child and adult victims). According to a recent meta-analysis, 19% of individuals who sexually offend were polymorphic, suggesting victim age polymorphism is somewhat common (Scurich & Gongola, 2021).

Previous research has often focused on the correlates of victim age polymorphism for the purpose of identifying potential risk factors for recidivism (e.g., Heil et al., 2003; Stephens et al., 2017). More broadly, atypical sexuality and antisociality are two overarching risk domains associated with sexual recidivism (Doren, 2004; Hanson & Morton-Bourgon, 2005). Previous research has suggested that those who offend against adults or children differ on these domains. Individuals with child victims tend to have elevated scores on measures of atypical sexuality (Paquette et al., 2022), which is in turn associated with sexual recidivism (Hanson & Morton-Bourgon, 2005). Individuals with adult victims tend to have elevated scores on measures of antisociality (Brown et al., 2015), which is in turn associated with violent recidivism (Hanson & Morton-Bourgon, 2005). It is possible that individuals who are polymorphic may show elevations in both domains; however, the associations between the risk-related domains and victim age polymorphism have been inconsistent in previous research (e.g., Brown et al., 2015; Cormier et al., 2020). There is a need to synthesize the literature on the risk-related domains and recidivism in those who are polymorphic, which is the focus of the present study.

### Atypical Sexuality

The association between atypical sexuality and victim selection has been the focus of extensive research, given that atypical sexuality is related to increased risk of sexual recidivism (e.g., Hanson & Morton-Bourgon, 2005; Mann et al., 2010; Seto et al., 2023). Atypical sexuality can be conceptualized as two separate constructs: paraphilias (persistent, intense, and sexual interest toward unusual targets, activities, or objects; American Psychiatric Association, 2022) and sexual preoccupation (an intense interest in sex that dominates psychological functioning; Hanson et al., 2007). Although these specific constructs are often emphasized, there are other related constructs that arguably fall into this domain (e.g., intense mating effort; Seto, 2019).

Laws (1994) argued that multiple paraphilias may motivate victim age polymorphism because multiple paraphilias are associated with a greater number of victims and sexual offenses. Nonetheless, studies that have explicitly examined the relationship between victim age polymorphism and multiple paraphilias have found no association (e.g., Cormier et al., 2020). Another possibility is that victim age polymorphism reflects a non-exclusive interest in different age groups, referred to as indiscriminate sexual arousal (Stephens et al., 2023). That is, victim age polymorphism may be associated with a lack of preference for a particular age, rather than a specific paraphilic interest. Relatively few studies have examined indiscriminate sexual arousal using phallometric devices, but there does appear to be some evidence to support its association with victim age polymorphism (Barbaree & Marshall, 1989; Michaud & Proulx, 2009; Stephens et al., 2023).

The association between sexual preoccupation and sexual recidivism has long been established (e.g., Cortoni & Marshall, 2001; Hanson & Morton-Bourgon, 2005). A study by Cormier and colleagues (2020) found that individuals who are polymorphic were no more likely to exhibit sexual preoccupation than those who are non-polymorphic, although it should be noted that the authors did not use a standardized measure of sexual preoccupation. Similarly, Lussier and colleagues (2007) suggested that individuals who are polymorphic may exhibit a higher degree of sexualization (i.e., a construct that captures a broad range of sexual behaviours including sexual preoccupation and paraphilias) than individuals who are non-polymorphic; however, the association between sexualization and polymorphism in the sample of 551 individuals was negligible (Lussier et al., 2007). Thus, while sexual preoccupation has been hypothesized to drive victim age polymorphism, an empirical basis for this claim has not yet been established.

### Antisociality

Much of the research on victim age polymorphism and antisociality has specifically examined psychopathy via the Psychopathy Checklist – Revised (PCL-R; Hare, 2003). Polymorphism has been found to be associated with elevated PCL-R scores in comparison with those who offend against child victims, those who offend against adult victims, and those with non-sexual offenses (medium to large associations; Brown et al., 2015; Jackson & Richards, 2007; Olver & Wong, 2006; Porter et al., 2000; Porter et al., 2001; Porter et al., 2009; Skovran et al., 2010). It should be noted that studies reporting elevated psychopathy scores among individuals who are polymorphic have small sample sizes, ranging from 19 (Skovran et al., 2010) to 56 individuals who are polymorphic (Jackson & Richards, 2007). A more recent study that included 105 individuals who were polymorphic found small, nonsignificant differences between sexual offending groups on the PCL-R; however, the authors reported lower-than-average psychopathy scores in a low risk (e.g., provincial treatment program) sample, which may have contributed to their findings (Cormier et al., 2020).

In addition to total PCL-R scores, researchers have examined differences in PCL-R Factor 1 and Factor 2 scores. Brown and colleagues (2015) found that individuals who are polymorphic had significantly higher Factor 1 scores than non-polymorphic individuals, while other studies have found that individuals who are polymorphic differed only from those with child victims (Porter et al., 2009; Skovran et al., 2010), or nonsignificant differences between all comparison groups (Cormier et al., 2020). Similarly, significantly higher scores on Factor 2 items have been found among individuals who are polymorphic only when compared with those with child victims (Brown et al., 2015; Nicholaichuk et al., 2000; Olver & Wong, 2006; Porter et al., 2009; Porter et al., 2000; see Cormier et al., 2020, for an exception). It is likely that the differences between individuals with child victims and those who are polymorphic are due to a significantly lower score on the part of those with child victims, rather than a significantly higher score on the part of those who are polymorphic, given that individuals with child victims also score lower on Factor 1 and 2 traits than those with adult victims and individuals with non-sexual offenses (Brown et al., 2015; Porter et al., 2000).

### Recidivism

Based on the above review of risk domains, individuals who are polymorphic appear similar to those who are non-polymorphic on atypical sexuality, but may score higher than individuals with child victims in the antisociality risk domain. These scores would be expected to translate into comparable rates of sexual recidivism and elevated rates of violent recidivism for those who are polymorphic. There is evidence of an elevated overall recidivism rate for those who are polymorphic in the literature (e.g., Harris et al., 2011; Link & Losel, 2021; Olver & Wong, 2006), although it is important to note that absolute probability estimates of recidivism can be unreliable across samples and settings (Helmus, 2021). Accordingly, the specific rates of sexual and violent recidivism among individuals who are polymorphic varies widely across studies.

In terms of sexual recidivism, individuals who are polymorphic have been found to demonstrate significantly higher sexual recidivism rates than those who target children and those who target adults (Harris et al., 2011; Olver & Wong, 2006; Parent et al., 2011), though other studies have found no difference between these groups (e.g., Link & Losel, 2021; Stephens et al., 2016). While the variation across studies may be due to sample size or characteristics, a possible methodological explanation for the variation is that the elevated sexual recidivism rate among those who are polymorphic may be driven by the number of victims, given that having multiple victims is associated with both polymorphism and recidivism (Hanson & Bussière, 1998; Helmus et al., 2015; Stephens et al., 2016). Several studies that reported elevated recidivism rates among those who are polymorphic did not exclude individuals with a single victim from the comparison groups (e.g., Harris et al., 2011; Olver & Wong, 2006; Parent et al., 2011). In contrast, Stephens and colleagues (2016) examined sexual recidivism in a sample that included 109 individuals with victim age polymorphic offenses and found that there was a significant association between victim age polymorphism and sexual recidivism; however, this association was no longer significant when controlling for the number of victims (Stephens et al., 2016). Thus, the variation in the relationship between recidivism and victim age polymorphism across studies may be explained by the inclusion of individuals with a single victim (Link & Losel, 2021; Stephens et al., 2016).

Rates of violent recidivism in the polymorphic group have been shown to differ significantly from individuals with child or adult victims (Link & Losel, 2021). Individuals who are polymorphic are generally found to have lower violent recidivism rates than individuals with adult victims and higher violent recidivism rates than individuals with child victims (Harris et al., 2011; Link & Losel, 2021; Olver & Wong, 2006; Parent et al., 2011; Vess & Skelton, 2010). Importantly, some of these studies have found very small differences in violent recidivism rates between those who are polymorphic and individuals with adult victims (e.g., Harris et al., 2011; Parent et al., 2011), which mirrors the results of the group differences in PCL-R scores (e.g., Skovran et al., 2010). Thus, previous research suggests that offending against adults, rather than victim age polymorphism, may drive the association with violent recidivism (Stephens et al., 2016). To inform this hypothesis, it would be beneficial to examine the association between violent recidivism and polymorphism.

### Methodological Considerations

The above-described literature highlights some variation in findings. It remains unclear whether this variation is due to true group differences or methodological artefacts. As previously discussed, sample characteristics such as risk level (e.g., Cormier et al., 2020; Porter et al., 2000) and the inclusion of individuals with a single victim (e.g., Stephens et al., 2016) may lead to inconsistent findings across studies. The source of offense information is another methodological factor that has been shown to influence results, as studies that use official records to determine offense history find lower rates of polymorphism than studies that use self-report methods (Scurich & Gongola, 2021).

Additionally, victim age polymorphism is conceptualized differently across studies in several important ways. First, researchers classify individuals with sexual offenses based on the age of their victim; however, different age cut-offs are used for the victim age categories. For example, one study may define a child as under age 11 (e.g., Stephens et al., 2017), while another defines a child as age 14 and under (e.g., Hanson et al., 2007). Second, the number of victim age groups differs as some studies may use two age categories (child and adult; e.g., Link & Losel, 2021), three age categories (child, adolescent, adult; e.g., Stephens et al., 2016), or four age categories (infant, prepubescent child, adolescent, adult; e.g., Cormier et al., 2020). The rate of polymorphism increases with the number of age groups in the study (Cormier et al., 2020; Saramago et al., 2018; Stephens et al., 2016), which may not reflect true polymorphism in situations where the victims appear similar in terms of sexual maturity. To date, the effect of different age categorization methods on various findings in the literature (e.g., association with psychopathy) has not been examined.

### Present Study

The present study used meta-analysis to examine the association between polymorphism and the overarching risk domains (atypical sexuality and psychopathy) and recidivism. A benefit of meta-analysis is that methodological differences across studies can be examined using meta-regression and between-level *Q*. We examined the impact of sample characteristics (e.g., risk-level, inclusion of single victim offenders), sources of information (official versus official and self-report), and victim age categorization (e.g., number of victim age categories, victim age cut offs) on study outcomes. The present study is important for advancing our understanding of clinically relevant factors among those who are polymorphic, as findings on risk-related domains and recidivism could impact the assessment, treatment, and supervision of individuals who are polymorphic.

## Method

This study was registered with PROSPERO (CRD42022345093), where a full copy of the original study protocol and prespecified analyses can be found. A review exemption was granted by the Saint Mary’s University Research Ethics Board (File #22-317) given that data were collected through publicly available sources. The authors take responsibility for the integrity of the data, the accuracy of the data analyses, and have made every effort to avoid inflating statistically significant results.

### Inclusion & Exclusion Criteria

Inclusion criteria for the meta-analysis were as follows: studies had to include a sample of adult men with sexual offense victims in multiple age categories, and report at least one measure of atypical sexuality (e.g., multiple paraphilias, sexual preoccupation, or similarly related construct), antisociality (e.g., PCL-R scores), and/or overall, sexual, or nonsexual violent recidivism. Only studies published in English (to ensure accuracy during data extraction) and those published after the year 2000 (due to the relative recency of victim age polymorphism research, improved operationalization of relevant constructs, and the need to contact authors for additional information) were included. Book chapters, systematic reviews, and meta-analyses were excluded given that they rarely present new data.

### Information Sources

Searches took place on April 18, 2022, November 12, 2022, and again on November 3, 2024. Studies were collected from PsycINFO/PsycArticles, PubMed, Web of Science, ProQuest, and Wiley Online Library. A compatible version of the following search string was used to obtain relevant study citations: (Sex Offen* OR Sexual Offen* OR Sexual Devian* OR Sex Devian* OR Sex Abuse OR Sexual Abuse) AND (Victim Polymorph* OR Polymorph* OR Mixed Offen* OR Victim Crossover OR Crossover OR Victim select* OR Victim Age). The online queues of *Sexual Abuse, Journal of Sexual Aggression,* and *Sexual Offending: Theory, Research and Prevention* were searched for pre-prints to ensure that the most recent data available was included. The studies included in Scurich and Gongola’s (2021) meta-analysis were also reviewed for inclusion given the topic relevance, and the reference lists of all studies that met the inclusion criteria were reviewed.

Grey literature, such as theses, dissertations, and conference abstracts were included in search parameters whenever possible. The Association for the Treatment & Prevention of Sexual Abuse website was searched using Victim Polymorph*, Mixed Offen*, Crossover, and Victim Age keywords for additional conference presentations. Thirteen authors who published polymorphism studies in the previous five years were contacted via email in April 2023 to provide any unpublished “file-drawer” studies. Authors were given a total of one month to respond to email inquiries, with a reminder email sent at the end of the second week. There was no further attempt to contact the authors after this reminder. The response rate to our requests for unpublished studies was 46% (*n* = 6 authors responded), and no file-drawer studies were provided by the authors. Therefore, publication bias was not examined in the present review.

### Study Selection

All studies that were identified in the study collection process were imported into Covidence and duplicate studies were removed. The first author and two undergraduate students screened abstracts for topic relevance, and two reviewers were required to vote on the topic relevance of each study. If the study was deemed relevant, it advanced to the full-text screening stage. At this stage, reviewers were instructed to read the methodology and results sections of each study and compare with the inclusion criteria. Studies advanced to the data extraction stage when two reviewers agreed that the inclusion criteria were met. Disagreements between reviewers during screening were flagged and the second and third authors were prompted to vote on the study to resolve the discrepancy.

Studies that met the inclusion criteria were examined by the first author for the purpose of identifying similar sample characteristics (e.g., same location, years of incarceration). Studies were collapsed and analyzed together in cases where samples completely overlapped, but the studies reported different variables. For partially overlapping samples that reported the same variables, only those that contained all the information necessary for analysis or the study with the largest sample size were used in the analysis. Table 1 reports the data extraction methods for studies with overlapping samples. The final list of included studies was compared to Cabells Predatory Reports to ensure that no studies were published in predatory journals.

**Table 1.** Characteristics of Studies Included in Meta-Analysis

Study	Sample information	Variables and analysis
Boughner (2010)	*N* = 232 (33 ISOVAPs; 148 ISOCs; 51 ISOAs) Men incarcerated for a sexual offense and released from Kentucky department of corrections (U.S.A.) between 1999 and 2000. Child victims were under the age of 18. Adult victims were 18 years or older.	Recidivism (sexual, nonsexual violent)
Brouillette-Alarie et al. (2018)	*N* = 558 (59 ISOVAPs; 355 ISOCs; 174 ISOAs) Men under federal supervision for contact sexual offense conviction(s) in Quebec (Canada) between 1995 and 2000. Child victims were under the age of 16. Adult victims were 16 years or older.	Multiple paraphilias (DSM-IV-TR) Sexual preoccupation (unique scale assembled for project) Recidivism (sexual, nonsexual violent)
Brown et al. (2015)	*N* = 719 (40 ISOVAPs; 211 ISOCs; 468 ISOAs) Men incarcerated for sexual offense conviction(s) in Wisconsin state prison (U.S.A.) between 2000 and 2013. Child victims were under the age of 18. Adult victims were 18 years or older.	Psychopathy (total PCL-R and factor scores)
Cormier et al. (2020)	*N* = 315 (34 ISOVAPs; 184 ISOCs; 97 ISOAs) Adult men with sexual offenses against 2+ victims assessed at a forensic outpatient clinic in Canada between 1998 and 2018. Reported multiple ISOC victim age groups (e.g., victims 0-5, 6-10, 11-14).	Multiple paraphilias (DSM version that was in use at the time of the assessment) Sexual preoccupation (clinician judgment) Psychopathy (total PCL-R and factor scores) For ISOC comparisons, the data for the 0-11 child victim age groups were combined (*n* = 110).
Ducro and Pham (2006)	*N* = 147 (17 ISOVAPs; 94 ISOCs; 36 ISOAs) Male patients in a high-security psychiatric hospital in Belgium who were deemed to lack the capacity to control their behaviour when sentenced for a sexual offense. Child victims were under the age of 14. Adult victims were 14 years or older.	Recidivism (overall, sexual, and nonsexual violent)
Howard et al. (2014)	*N* = 1,586 (464 ISOVAPs; 712 ISOCs; 410 ISOAs) Men with a criminal record for sexual offending per the offender assessment system in England & Wales. Victim age operationalization is unknown.	Recidivism (overall, sexual, nonsexual violent)
Langevin et al. (2004)	*N* = 250 (28 ISOVAPs; 188 ISOCs; 34 ISOAs) Men referred for assessment or treatment in relation to a sexual offense in Canada between 1966 and 1974. Victim age operationalization is unknown.	Recidivism (overall, sexual)
Link and Losel (2021)	*N* = 508 (85 ISOVAPs; 287 ISOCs; 136 ISOAs) Men sentenced to 2+ years in prison for a sexual index offense and released from Bavarian prisons (Germany) between January 2004 and June 2015. Child victims were under the age of 14. Adult victims were 14 years or older.	Recidivism (overall, sexual, nonsexual violent)
Michaud and Proulx (2009)	*N* = 466 (29 ISOVAPs; 263 ISOCs; 174 ISOAs) Adult men undergoing assessment while in presentence detainment or a maximum security forensic inpatient clinic related to a sexual offense in Canada. Child victims were under the age of 14. Adult victims were 14 years or older.	Indiscriminate arousal (Circumferential assessment of max response to various stimuli according to victim age and paraphilia) One of two studies that reported data on indiscriminate arousal, but this study reported paraphilic information alongside victim age preference. Therefore, data could not be synthesized.
Olver and Wong (2006)	*N* = 127 (26 ISOVAPs; 25 ISOCs; 76 ISOAs) Adult men serving federal sentences for a sexual offense who were admitted to the Clearwater sex offender treatment program in Canada between 1983 and 1997. Child victims were under the age of 14. Adult victims were 14 years or older.	Psychopathy (total PCL-R and factor scores) Recidivism (sexual, nonsexual violent)
Parent et al. (2011)	*N* = 503 (54 ISOVAPs; 275 ISOCs; 174 ISOAs) Men evaluated at the Massachusetts treatment center for sexually dangerous persons in the U.S.A. between 1959 and 1984. Child victims were under the age of 16. Adult victims were 16 years or older.	Psychopathy (total PCL-R scores) Recidivism (sexual, nonsexual violent)
Porter et al. (2000)	*N* = 229 (25 ISOVAPs; 88 ISOCs; 103 ISOAs) Men serving a federal sentence for a sexual offense in Canada. Child victims were under the age of 15. Adult victims were 15 years or older.	Psychopathy (total PCL-R and factor scores) This study reported the ISOC group separated by intrafamilial and extrafamilial victims. The same sample was used in a later publication (Porter et al., 2009) with the intrafamilial and extrafamilial ISOCs combined. Therefore, ISOC data was extracted from Porter et al. (2009).
Ryan et al. (2017)	*N* = 293 (51 ISOVAPs; 209 ISOCs; 33 ISOAs) Men convicted of a sexual offense and held in a state forensic mental health hospital in the U.S.A. Victim age operationalization is unknown.	Sexual preoccupation (sexual compulsivity scale) This study used the same sample as Skovran et al. (2010). Both of these studies reported data on sexual preoccupation, which was extracted from Ryan et al. (2017) given its greater sample size.
Skovran et al. (2010)	*N* = 134 (19 ISOVAPs; 95 ISOCs; 20 ISOAs) Men convicted of a sexual offense and held in a state forensic mental health hospital in the U.S.A. Child victims were under the age of 18. Adult victims were 18 years or older.	Psychopathy (total PCL-R and factor scores) This study used the same sample as Ryan et al. (2017), which had a greater sample size. This study was also included in the analysis because it reported psychopathy data (Ryan et al., 2017, did not).
Stephens et al. (2017)	*N* = 751 (168 ISOVAPs; 460 ISOCs; 123 ISOAs) Men referred for assessment at a sexual behaviour clinic in Canada. Child victims were under the age of 15. Adult victims were 15 years or older.	Recidivism (sexual)
Stephens et al. (2023)	*N* = 2,411 (1,787 ISOVAPs; 107 ISOCs; 834 ISOAs) Men referred for assessment at a sexual behaviour clinic in Canada between 1995 and 2011. Child victims were under the age of 15. Adult victims were 15 years or older.	Indiscriminate arousal (Volumetric assessment of pedohebephilia index and max response to victim age group) One of two studies that reported data on indiscriminate arousal, but this study reported only on victim age preference. Therefore, data could not be synthesized.
Stinson et al. (2008)	*N* = 95 (18 ISOVAPs; 59 ISOCs; 18 ISOAs) Men civilly committed in the U.S.A. Victim age operationalization is unknown.	Multiple paraphilias (Multiphasic sex Inventory-II) Psychopathy (total PCL-R and factor scores)
Stinson et al. (2017)	*N* = 156 (52 ISOVAPs; 48 ISOCs; 56 ISOAs) Men eligible for sex offense treatment services due to history of sexual offending or sexually problematic behaviour in the correctional facility in the U.S.A. Victim age operationalization is unknown.	Multiple paraphilias (various diagnoses)
Vess and Skelton (2010)	*N* = 2,435 (402 ISOVAPs; 1,165 ISOCs; 868 ISOAs) Men sentenced for a sexual offense who were released from prison in New Zealand between 1990 and 1995. Child victims were under the age of 16. Adult victims were 16 years or older.	Recidivism (sexual)
Walters et al. (2016)	*N* = 287 (99 ISOVAPs; 188 ISOCs) Men incarcerated for a sexual offense who were assessed upon entering a federal prison in Canada Child victims were under the age of 16. Adult victims were 16 years or older.	Psychopathy (total PCL-R and factor scores) Only ISOCs were used as a comparison group during analyses. One comparison group was not used because it was a combination of ISOs with various victim ages (e.g., ISOVAPs, ISOAs, and individuals who offended against teens)
Woodworth et al. (2013)	*N* = 101 (18 ISOVAPs; 41 ISOCs; 42 ISOAs) High-risk men incarcerated for a sexual offense in Canada. Child victims were under the age of 14. Adult victims were 14 years or older.	Multiple paraphilias (DSM-IV-TR)
Zanatta (2005) ^,^	*N* = 164 (40 ISOVAPs; 34 ISOCs; 90 ISOAs) equally divided into two matched samples. Zanatta (2005a): Men convicted of a sexual offense and designated as a dangerous offender in Canada between 1978 and 2002. Zanatta (2005b): Men convicted of 2+ sexual offenses against multiple victims from the pacific region of the Correctional service of Canada.	Psychopathy (total PCL-R and factor scores) This study reported ISOVAP, ISOC and ISOA comparisons across two different samples (reported as Zanatta, 2005a and 2005b).

*Note. k* = 22, representing 23 unique samples. ISOVAPs = Individuals with sexual offenses that are victim age polymorphic; ISOCs = Individuals with sexual offenses against only children; ISOAs = Individuals with sexual offenses against only adults; PCL-R = Psychopathy Checklist-Revised.

### Data Extraction

The first and second authors extracted relevant data from each study independently before meeting to obtain consensus. All projects were coded for variables in four broad categories: study information (e.g., authors, year), sample characteristics (e.g., age, ethnicity, sample setting), methodology variables (e.g., victim age classification methods, sample risk level), and outcome variables (e.g., operationalization and effect size information for risk-related domains and recidivism).

In some cases, authors were contacted to provide the data necessary to calculate effect sizes (e.g., missing standard deviations). Twenty-seven corresponding authors were contacted to provide missing data. Just under half of the authors responded to these data requests (44%; *n* = 12), three of whom (11%) were unable to provide the data due to access restrictions. After data extraction was completed, a second and third round of searches were conducted to capture newly published studies, and the screening and extraction processes were repeated. The extracted consensus data were then imported into SPSS for analysis.

### Summary Measures

Synthesis was performed using two summary measures: Cohen’s *d* and Log Risk Ratio (*RR*). For continuous variables, Cohen’s *d* was calculated using the mean, standard deviation, and sample size for each group. For categorical variables, Log Risk Ratios were calculated using the number of individuals with sexual offenses that met a certain condition compared with those who did not (e.g., diagnosed with multiple paraphilias versus diagnosed with less than two paraphilias; recidivists versus non-recidivists). One study reported continuous data for a measure of sexual preoccupation while all others reported categorical data. In this case, the Odds Ratio for the categorical effect size was converted to Cohen’s *d* for analysis using DeCoster's (2012) effect size converter. The standard error was calculated by dividing the Log Odds Ratio by 1.65 to convert to the Cohen’s *d* distribution (Sanchez-Meca et al., 2003).

### Methods of Synthesis

Deviating slightly from our preregistration plan, weighted means for each outcome variable were calculated using both inverse-variance random-effects and fixed-effects models. Random-effects models are used when the effect sizes reported vary across studies based on more than just sampling error (e.g., methodological variables), which can be measured by examining heterogeneity of the data (Hanson, 2022). Heterogeneity was calculated using the *I*^2^ statistic, which can be used to classify the magnitude of heterogeneity (i.e., 25% = low; 50% = moderate; 75% = high; Higgins & Green, 2011). If heterogeneity was low (*I*^2^ < 25%), or in cases where there was a small number of studies synthesized, a fixed-effects model may be more appropriate for the data. The Hartung-Knapp correction was applied to random-effects analyses given recent recommendations for widespread use (van Aert & Jackson, 2019).

Moderator variables included victim age categorization methods (number of categories, victim age cutoffs), sample characteristics (single-victim inclusion, risk level), and source of information (official versus both official and self-report). Sample risk was determined using the Static-99R manual when Static-99R scores were reported in the study. If studies did not report risk using an actuarial risk tool, sample risk was estimated using sample setting. For example, samples from civil commitment centers or high-risk treatment programs were coded as high risk. The effect of these moderators on each outcome variable was estimated using the between-level *Q* statistic (categorical moderators) and meta-regression (continuous moderators). Like multiple regression, meta-regression is used to assess the relationship between a dependent variable (effect size) and covariates (moderators; Borenstein et al., 2009). It is generally recommended that meta-regression be performed when there is high heterogeneity and at least ten studies included in the analyses (Borenstein et al., 2009).

## Results

### Study Selection

**Figure 1.**
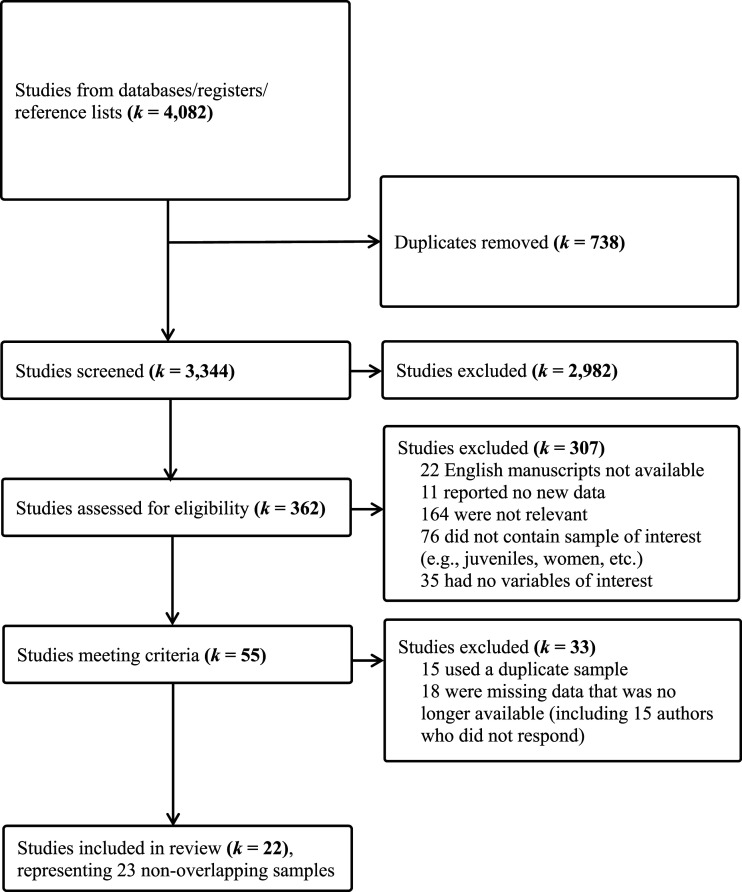
PRISMA diagram. *Note.* PRISMA = Preferred Reporting Items for Systematic reviews and Meta-Analyses

The first round of study collection yielded 3,455 articles across all databases (2,774 articles after removing duplicates), the second round yielded 140 articles (131 without duplicates), and the third round yielded 466 articles (418 without duplicates). An additional 21 studies were identified as potentially relevant by reviewing the reference lists of included studies. Therefore, a total of 3,344 studies were screened for inclusion. More detailed information about study collection and screening can be found in the Preferred Reporting Items for Systematic reviews and Meta-Analyses (PRISMA) diagram (Figure 1).

The interrater reliability for the abstract screening stage was moderate to substantial, κ = .61, with 93% agreement across reviewers. The interrater reliability for the full-text screening stage was substantial, κ = .79, with 97% agreement across reviewers. In the data extraction stage, a total of 28 out of 93 effect sizes were identified by one coder but not the other, and an additional 11 effect sizes were provided by the authors we contacted.

### Study Characteristics

Twenty-three samples from 22 studies were included in the analyses (Zanatta, 2005, contained two samples).^1^ Five studies reported data on multiple paraphilias, three studies examined sexual preoccupation, 11 studies examined psychopathy, and 10 studies reported recidivism data. Table 1 includes study characteristics, extraction and analysis information.

### Meta-Analytic Synthesis

Both fixed- and random-effects models are reported below due to the small number of synthesized studies. The effect sizes for all included studies can be found in Table 2 (multiple paraphilias), Table 3 (sexual preoccupation), Table 4 (antisociality), and Table 5 (recidivism), alongside the results of each synthesis. Forest plots are available in the Supplemental Material.

**Table 2.** Individual Effect Sizes for Multiple Paraphilia Studies

ISOVAP comparison	Study	Fixed-effects model		Random-effects model	
		Risk ratio [95% CI]	Weight (%)	Risk ratio [95% CI]	Weight (%)
ISOC	Stinson et al. (2008)	−0.003 [−0.47, 0.47]	14.41	−0.003 [−0.47, 0.47]	21.34
	Woodworth et al. (2013)	0.59 [−1.46, 2.63]	0.76	0.59 [−1.46, 2.63]	1.32
	Stinson et al. (2017)	2.30 [−0.57, 5.17]	0.39	2.30 [−0.57, 5.17]	0.67
	Brouillette-Alarie et al. (2018)	0.34 [0.14, 0.54]	80.35^a^	0.34 [0.14, 0.54]	69.85^b^
	Cormier et al. (2020)	0.002 [−0.88, .88]	4.10	0.002 [−0.88, 0.88]	6.81
	Total log risk ratio [95% CI]	0.28 [0.11, 0.46], *I*^*2*^ = 2.2%		0.26 [−0.06, 0.58], *I*^*2*^ = 9.5%	
ISOA	Stinson et al. (2008)	0.80 [−0.15, 1.74]	8.31	0.80 [−0.15, 1.74]	19.68
	Woodworth et al. (2013)	−0.51 [−1.58, 0.55]	6.53	−0.51 [−1.58, 0.55]	17.56
	Stinson et al. (2017)	0.95 [−0.64, 2.55]	2.91	0.95 [−0.64, 2.55]	10.82
	Brouillette-Alarie et al. (2018)	1.08 [0.77, 1.40]	73.92^c^	1.08 [0.77, 1.40]	32.28
	Cormier et al. (2020)	0.02 [−0.93, 0.96]	8.32	0.02 [−0.93, 0.96]	19.67
	Total log risk ratio [95% CI]	0.86 [0.59, 1.13], *I*^*2*^ = 64.7%		0.52 [−0.33, 1.37], *I*^*2*^ = 62.4%	

*Note. k* = 5. ISOC = Individuals with sexual offenses against children; ISOA = Individuals with sexual offenses against adults; ISOVAP = Individuals with sexual offenses that are victim age polymorphic. Positive risk ratios indicate ISOVAPs had increased likelihood of multiple paraphilias. Negative risk ratios indicate ISOVAPs had decreased likelihood of multiple paraphilias.

^a^Study is an outlier due to large weight. Excluding the outlier, the effect size was negligible in the fixed-effect analysis, log *RR* = 0.07, 95% CI[−0.34, 0.47], *I*^*2*^ = 0.0%.

^b^Study is an outlier due to large weight. Excluding the outlier, the effect size was negligible in the fixed-effects analysis, log *RR* = 0.07, 95% CI[−0.56, 0.69], *I*^*2*^ = 0.5%.

^c^Study is an outlier due to large weight. Excluding the outlier, the effect size in the random-effects analysis was small, log *RR* = 0.24, 95% CI[−0.29, 0.77], *I*^*2*^ = 29.1%.

**Table 3.** Individual Effect Sizes for Sexual Preoccupation Studies

ISOVAP comparison	Study	Fixed-effects model		Random-effects model	
		Cohen’s *d* [95% CI]	Weight (%)	Cohen’s *d* [95% CI]	Weight (%)
ISOC	Ryan et al. (2017)	0.12 [−0.21, 0.44]	32.87	0.12 [−0.21, 0.45]	32.87
	Brouillette-Alarie et al. (2018)	0.19 [−0.08, 0.46]	48.47	0.19 [−0.08, 0.46]	48.47
	Cormier et al. (2020)	−0.06 [−0.50, 0.39]	18.67	−0.06 [−0.50, 0.39]	18.66
	Total Cohen’s *d* [95% CI]	0.12 [−0.07, 0.31], *I*^*2*^ = 0.0%		0.12 [−0.15, 0.40], *I*^*2*^ = 0.0%	
ISOA	Ryan et al. (2017)	0.81 [0.30, 1.32]	19.29	0.81 [0.30, 1.32]	30.90
	Brouillette-Alarie et al. (2018)	−0.08 [−0.37, 0.21]	57.94^a^	−0.08 [−0.37, 0.21]	36.98
	Cormier et al. (2020)	−0.12 [−0.59, 0.35]	22.77	−0.12 [−0.59, 0.35]	32.11
	Total Cohen’s *d* [95% CI]	0.08 [−0.14, 0.31], *I*^*2*^ = 79.4%		0.18 [−1.09, 1.46], *I*^*2*^ = 82.0%	

*Note. k* = 3. ISOC = Individuals with sexual offenses against children; ISOA = Individuals with sexual offenses against adults; ISOVAP = Individuals with sexual offenses that are victim age polymorphic. Positive Cohen’s *d* indicates increased association between sexual preoccupation and victim age polymorphism. Negative Cohen’s *d* indicates decreased association between sexual preoccupation and victim age polymorphism.

^a^Study is an outlier due to large weight. Excluding the outlier, the fixed-effects analysis revealed a small effect size, *d* = 0.31, 95% CI[−0.04, 0.65], *I*^2^ = 85.5%.

**Table 4.** Individual Effect Sizes for PCL-R Studies

Variable	ISOVAP comparison	Study	Fixed-effects model		Random-effects model	
			Cohen’s *d* [95% CI]	Weight (%)	Cohen’s *d* [95% CI]	Weight (%)
PCL-R total scores	ISOC (*k* = 10)	Porter et al. (2000)	1.24 [0.76, 1.71]	7.18	1.24 [0.76, 1.71]	9.82
		Zanatta (2005a)	0.71 [0.05, 1.38]	3.61	0.71 [0.05, 1.38]	7.10
		Zanatta (2005b)	0.65 [−0.02, 1.31]	3.64	0.65 [−0.02, 1.31]	7.14
		Olver and Wong (2006)	1.03 [0.45, 1.61]	4.70	1.03 [0.45, 1.61]	8.15
		Stinson et al. (2008)	0.17 [−0.36, 0.69]	5.78	0.17 [−0.36, 0.69]	8.98
		Skovran et al. (2010)	0.66 [0.16, 1.16]	6.41	0.66 [0.16, 1.16]	9.39
		Parent et al. (2011)	0.44 [0.15, 0.74]	18.58	0.44 [0.15, 0.74]	12.89
		Brown et al. (2015)	0.35 [0.01, 0.69]	13.91	0.35 [0.01, 0.69]	12.09
		Walters et al. (2016)	0.05 [−0.20, 0.29]	27.05	0.05 [−0.20, 0.29]	13.73
		Cormier et al. (2020)	0.35 [−0.07, 0.77]	9.14	0.35 [−0.07, 0.77]	10.71
		Total Cohen’s *d* [95% CI]	0.41 [0.29, 0.54], *I*^2^ = 67.7%		0.52 [0.25, 0.79], *I*^2^ = 67.5%	
	ISOA (*k* = 9)	Porter et al. (2000)	0.51 [0.07, 0.96]	10.63	0.51 [0.07, 0.96]	11.51
		Zanatta (2005a)	−0.19 [−0.72, 0.34]	7.44	−0.19 [−0.72, 0.34]	10.71
		Zanatta (2005b)	−0.69 [−1.23, −0.15]	7.11	−0.69 [−1.23, −0.15]	10.59
		Olver and Wong (2006)	0.11 [−0.34, 0.56]	10.44	0.11 [−0.34, 0.56]	11.47
		Stinson et al. (2008)	−1.19 [−1.92, −0.46]	3.89	−1.19 [−1.92, −0.46]	8.84
		Skovran et al. (2010)	0.03 [−0.60, 0.66]	5.26	0.03 [−0.60, 0.66]	9.77
		Parent et al. (2011)	−0.13 [−0.44, 0.17]	22.20	−0.13 [−0.44, 0.17]	12.65
		Brown et al. (2015)	0.72 [0.39, 1.04]	19.52	0.72 [0.39, 1.04]	12.50
		Cormier et al. (2020)	0.24 [−0.15, 0.64]	13.51	0.24 [−0.15, 0.64]	11.95
		Total Cohen’s *d* [95% CI]	0.10 [−0.04, 0.25], *I*^2^ = 80.7%		−0.02 [−0.46, 0.41], *I*^2^ = 82.7%	
PCL-R factor 1 scores	ISOC (*k* = 9)	Porter et al. (2000)	0.59 [0.14, 1.04]	10.22	0.59 [0.14, 1.04]	11.48
		Zanatta (2005a)	0.48 [−0.18, 1.13]	4.83	0.48 [−0.18, 1.13]	7.12
		Zanatta (2005b)	0.13 [−0.52, 0.77]	4.96	0.13 [−0.52, 0.77]	7.25
		Olver and Wong (2006)	0.26 [−0.29, 0.81]	6.83	0.26 [−0.29, 0.81]	9.02
		Stinson et al. (2008)	0.14 [−0.39, 0.67]	7.50	0.14 [−0.39, 0.67]	9.57
		Skovran et al. (2010)	0.52 [0.03, 1.02]	8.40	0.52 [0.03, 1.02]	10.26
		Brown et al. (2015)	0.74 [0.39, 1.08]	17.54	0.74 [0.39, 1.08]	14.89
		Walters et al. (2016)	−0.02 [−0.30, 0.26]	26.53	−0.02 [−0.30, 0.26]	17.32
		Cormier et al. (2020)	0.18 [−0.22, 0.58]	13.17	0.18 [−0.22, 0.58]	13.09
		Total Cohen’s *d* [95% CI]	0.31 [0.16, 0.45], *I*^*2*^ = 46.3%		0.33 [0.12, 0.54], *I*^*2*^ = 46.1%	
	ISOA (*k* = 8)	Porter et al. (2000)	0.50 [0.06, 0.94]	13.55	0.50 [0.06, 0.94]	13.49
		Zanatta (2005a)	0.19 [−0.34, 0.72]	9.48	0.19 [−0.34, 0.72]	11.86
		Zanatta (2005b)	−0.50 [−1.03, 0.04]	9.27	−0.50 [−1.03, 0.04]	11.76
		Olver and Wong (2006)	0.03 [−0.42, 0.47]	13.31	0.03 [−0.42, 0.47]	13.41
		Stinson et al. (2008)	−0.86 [−1.56, −0.15]	5.33	−0.86 [−1.56, −0.15]	9.05
		Skovran et al. (2010)	−0.10 [−0.72, 0.53]	6.69	−0.10 [−0.72, 0.53]	10.17
		Brown et al. (2015)	0.36 [0.04, 0.69]	25.20	0.36 [0.04, 0.69]	15.81
		Cormier et al. (2020)	0.29 [−0.10, 0.68]	17.16	0.29 [−0.10, 0.68]	14.45
		Total Cohen’s *d* [95% CI]	0.13 [−0.03, 0.30], *I*^*2*^ = 63.0%		0.05 [−0.31, 0.41], *I*^*2*^ = 65.6%	
PCL-R factor 2 scores	ISOC (*k* = 9)	Porter et al. (2000)	1.08 [0.61, 1.54]	9.22	1.08 [0.61, 1.54]	11.19
		Zanatta (2005a)	0.37 [−0.28, 1.02]	4.71	0.37 [−0.28, 1.02]	7.90
		Zanatta (2005b)	0.51 [−0.15, 1.17]	4.64	0.51 [−0.15, 1.17]	7.83
		Olver and Wong (2006)	1.03 [0.44, 1.61]	5.87	1.03 [0.44, 1.61]	8.96
		Stinson et al. (2008)	0.28 [−0.25, 0.81]	7.19	0.28 [−0.25, 0.81]	9.97
		Skovran et al. (2010)	0.66 [0.16, 1.16]	8.01	0.66 [0.16, 1.16]	10.50
		Brown et al. (2015)	0.53 [0.19, 0.87	17.20	0.53 [0.19, 0.87]	14.01
		Walters et al. (2016)	0.28 [.001, 0.56]	25.34	0.28 [.001, 0.56]	15.46
		Cormier et al. (2020)	0.03 [−0.30, 0.37]	17.82	0.03 [−0.30, 0.37]	14.16
		Total Cohen’s *d* [95% CI]	0.44 [0.30, 0.58], *I*^*2*^ = 58.9%		0.50 [0.23, 0.77], *I*^*2*^ = 59.9%	
	ISOA (*k* = 8)	Porter et al. (2000)	0.07 [−0.37, 0.51]	12.49	0.07 [−0.37, 0.51]	13.40
		Zanatta (2005a)	−0.40 [−0.93, 1.32]	8.46	−0.40 [−0.93, 1.32]	11.61
		Zanatta (2005b)	−0.65 [−1.19, −0.11]	8.23	−0.65 [−1.19, −0.11]	11.48
		Olver and Wong (2006)	0.08 [−0.37, 0.52]	12.02	0.08 [−0.37, 0.52]	13.23
		Stinson et al. (2008)	−1.04 [−1.76, −0.33]	4.63	−1.04 [−1.76, −0.33]	8.65
		Skovran et al. (2010)	0.28 [−0.35, 0.91]	5.99	0.28 [−0.35, 0.91]	9.92
		Brown et al. (2015)	0.26 [−0.06, 0.59]	22.83	0.26 [−0.06, 0.59]	15.69
		Cormier et al. (2020)	−0.19 [−0.49, 0.12]	25.35	−0.19 [−0.49, 0.12]	16.02
		Total Cohen’s *d* [95% CI]	−0.09 [−0.24, 0.07], *I*^*2*^ = 64.2%		−0.15 [−0.51, 0.21], *I*^*2*^ = 66.6%	

*Note.* ISOC = Individuals with sexual offenses against children; ISOA = Individuals with sexual offenses against adults; ISOVAP = Individuals with sexual offenses that are victim age polymorphic; PCL-R = Psychopathy Checklist-Revised. Positive Cohen’s *d* indicates increased association between PCL-R scores and victim age polymorphism. Negative Cohen’s *d* indicates decreased association between PCL-R scores and victim age polymorphism. No outliers were found in any of the syntheses.

**Table 5.** Individual Effect Sizes for Recidivism Studies

Variable	ISOVAP comparison	Study	Fixed-effects model		Random-effects model	
			Risk ratio [95% CI]	Weight (%)	Risk ratio [95% CI]	Weight (%)
Overall recidivism (k = 4)	ISOC	Langevin et al. (2004)	−0.05 [−0.23, 0.14]	34.76	−0.05 [−0.23, 0.14]	30.23
		Ducro and Pham (2006)	−0.13 [−0.77, 0.51]	2.92	−0.13 [−0.77, 0.51]	12.09
		Howard et al. (2014)	0.27 [0.11, 0.43]	45.30	0.27 [0.11, 0.43]	31.23
		Link and Losel (2021)	0.51 [0.24, 0.77]	17.03	0.51 [0.24, 0.77]	26.44
		Total risk ratio [95% CI]	0.19 [0.08, 0.30], *I*^*2*^ = 77.9%		0.19 [−0.25, 0.63], *I*^*2*^ = 79.5%	
	ISOA	Langevin et al. (2004)	−0.14 [−0.33, 0.06]	32.20	−0.14 [−0.33, 0.06]	33.41
		Ducro and Pham (2006)	0.33 [−0.52, 1.17]	1.68	0.33 [−0.52, 1.17]	2.43
		Howard et al. (2014)	−0.21 [−0.36, −0.06]	50.05	−0.21 [−0.36, −0.06]	44.60
		Link and Losel (2021)	0.06 [−0.21, 0.33]	16.07	0.06 [−0.21, 0.33]	19.57
		Total risk ratio [95% CI]	−0.13 [−0.24, -0.02], *I*^*2*^ = 24.8%		−0.12 [−0.34, 0.10], *I*^*2*^ = 21.3%	
Sexual recidivism (k = 10)	ISOC	Langevin et al. (2004)	−0.15 [−0.35, 0.05]	41.02	−0.15 [−0.35, 0.05]	16.39
		Ducro and Pham (2006)	−0.05 [−0.84, 0.75]	2.58	−0.05 [−0.84, 0.75]	7.06
		Olver and Wong (2006)	0.37 [−0.34, 1.07]	3.28	0.37 [−0.34, 1.07]	8.11
		Boughner (2010)	0.40 [−0.85, 1.65]	1.05	0.40 [−0.85, 1.65]	3.73
		Vess and Skelton (2010)	0.46 [0.19, 0.73]	23.13	0.46 [0.19, 0.73]	15.33
		Parent et al. (2011)	0.89 [0.49, 1.30]	10.11	0.89 [0.49, 1.30]	12.89
		Howard et al. (2014)	−0.01 [−0.59, 0.57]	4.86	−0.01 [−0.59, 0.57]	9.87
		Brouillette-Alarie et al. (2018)	0.80 [0.13, 1.47]	3.63	0.80 [0.13, 1.47]	8.56
		Stephens et al. (2017)	0.26 [−0.19, 0.70]	8.40	0.26 [−0.19, 0.70]	12.19
		Link and Losel (2021)	−0.38 [−1.30, 0.54]	1.94	−0.38 [−1.30, 0.54]	5.87
		Total risk ratio [95% CI]	0.19 [0.06, 0.32], *I*^*2*^ = 72.6%		0.28 [−0.01, 0.57], *I*^*2*^ = 67.7%	
	ISOA	Langevin et al. (2004)	−0.08 [−0.32, 0.16]	33.99	−0.08 [−0.32, 0.16]	15.36
		Ducro and Pham (2006)	0.75 [−0.35, 1.85]	1.61	0.75 [−0.35, 1.85]	5.38
		Olver and Wong (2006)	0.23 [−0.28, 0.73]	7.47	0.23 [−0.28, 0.73]	11.58
		Boughner (2010)	0.15 [−1.28, 1.58]	0.94	0.15 [−1.28, 1.58]	3.64
		Vess and Skelton (2010)	0.27 [0, 0.54]	26.51	0.27 [0.00, 0.54]	14.97
		Parent et al. (2011)	0.81 [0.37, 1.25]	10.17	0.81 [0.37, 1.25]	12.64
		Howard et al. (2014)	−0.70 [−1.26, −0.14]	6.12	−0.70 [−1.26, −0.14]	10.83
		Brouillette-Alarie et al. (2018)	0.34 [−0.35, 1.03]	4.03	0.34 [−0.35, 1.03]	9.12
		Stephens et al. (2017)	−0.35 [−0.85, 0.15]	7.82	−0.35 [−0.85, 0.15]	11.75
		Link and Losel (2021)	0.39 [−0.82, 0.15]	1.33	0.39 [−0.82, 0.15]	4.73
		Total risk ratio [95% CI]	0.11 [−0.03, 0.24], *I*^*2*^ = 66.8%		0.13 [−0.20, 0.47], *I*^*2*^ = 71.7%	
Violent recidivism (k = 7)	ISOC	Ducro and Pham (2006)	0.88 [0.16, 1.61]	7.00	0.88 [0.16, 1.61]	11.15
		Olver and Wong (2006)	1.26 [0.11, 2.41]	2.75	1.26 [0.11, 2.41]	5.12
		Boughner (2010)	0.02 [−0.88, 0.91]	4.57	0.02 [−0.88, 0.91]	7.92
		Parent et al. (2011)	0.20 [−0.36, 0.76]	11.72	0.20 [−0.36, 0.76]	16.10
		Howard et al. (2014)	0.54 [0.29, 0.79]	56.62^a^	0.54 [0.29, 0.79]	33.67^b^
		Brouillette-Alarie et al. (2018)	0.56 [−0.06, 1.17]	9.76	0.56 [−0.06, 1.17]	14.22
		Link and Losel (2021)	1.31 [0.61, 2.00]	7.57	1.31 [0.61, 2.00]	11.83
		Total risk ratio [95% CI]	0.58 [0.39, 0.77], *I*^*2*^ = 37.4%		0.61 [0.23, 0.99], *I*^*2*^ = 32.2%	
	ISOA	Ducro and Pham (2006)	1.31 [0.23, 2.40]	2.52	1.31 [0.23, 2.40]	2.52
		Olver and Wong (2006)	−0.14 [−0.65, 0.36]	11.63	−0.14 [−0.65, 0.36]	11.64
		Boughner (2010)	−0.73 [−1.63, 0.18]	3.63	−0.73 [−1.63, 0.18]	3.63
		Parent et al. (2011)	−0.47 [−1.01, 0.07]	10.25	−0.47 [−1.01, 0.07]	10.26
		Howard et al. (2014)	−0.16 [−0.40, 0.07]	53.99^c^	−0.16 [−0.40, 0.07]	53.95^d^
		Brouillette-Alarie et al. (2018)	−0.49 [−1.07, 0.09]	8.85	−0.49 [−1.07, 0.09]	8.86
		Link and Losel (2021)	−0.31 [−0.88, 0.26]	9.13	−0.31 [−0.88, 0.26]	9.14
		Total risk ratio [95% CI]	−0.22 [−0.39, -0.05], *I*^*2*^ = 45.3%		−0.22 [−0.51, 0.07], *I*^*2*^ = 0.0%	

*Note.* ISOC = Individuals with sexual offenses against children; ISOA = Individuals with sexual offenses against adults; ISOVAP = Individuals with sexual offenses that are victim age polymorphic. Positive risk ratios indicate ISOVAPs had increased recidivism. Negative risk ratios indicate ISOVAPs had decreased recidivism.

^a^Study is an outlier due to large weight. Excluding the outlier, the effect size in the fixed-effects synthesis remained medium, log *RR* = 0.63, 95% CI[0.34, 0.92], *I*^*2*^ = 46.6%.

^b^Study is an outlier due to large weight. Excluding the outlier, the effect size in the random-effects synthesis remained medium, log *RR* = 0.66, 95% CI[0.12, 1.20], *I*^*2*^ = 46.5%.

^c^Study is an outlier due to large weight. Excluding the outlier, the effect size in the fixed-effects synthesis remained small, log *RR* = −0.28, 95% CI[−0.54, −0.03], *I*^*2*^ = 52.4%.

^d^Study is an outlier due to large weight. Excluding the outlier, the effect size in the random-effects synthesis remained small, log *RR* = −0.28, 95% CI[−0.77, 0.21], *I*^*2*^ = 5.1%.

Due to the limited number of studies, meta-regression could only be performed for PCL-R total scores (only the polymorphic and child victim comparison; the adult victim comparison had just nine studies) and sexual recidivism analyses using victim age cutoff (according to highest age of child group) as the moderator. The average follow up period of the studies was also included as a moderator for the sexual recidivism analyses given that most studies did not perform analyses that accounted for differences in follow up times (e.g., Cox regression). For categorical moderators, we were only able to examine the impact of information source (official only versus both official and self-report) on the psychopathy (total, Factor 1, and Factor 2 scores) and recidivism (sexual and violent) analyses. The remaining between-level *Q* statistics could not be calculated because there was very little variation in the categorical moderators (i.e., sample risk level, exclusion of individuals with a single victim) between studies. A summary of the moderator analyses can be found in Tables 6 (meta-regression) and 7 (between-level *Q*).

**Table 6.** Meta-regression Analyses

Variable	Moderator	ISOVAP comparison	Fixed-effects model			Random-effects model		
			*b*	*SE*	*p*	*b*	*SE*	*p*
Total PCL-R	Victim age	ISOC	0.02	0.04	.75	0.01	0.06	.84
Sexual recidivism	Victim age	ISOC	0.27	0.13	.14	0.27	0.13	.13
		ISOA	0.12	0.13	.44	0.11	0.12	.43
	Follow up time	ISOC	−0.09	0.05	.17	−0.09	0.05	.16
		ISOA	−0.08	0.05	.21	−0.08	0.05	.20

*Note.* ISOC = Individuals with sexual offenses against children; ISOA = Individuals with sexual offenses against adults; ISOVAP = Individuals with sexual offenses that are victim age polymorphic; PCL-R = Psychopathy Checklist-Revised. Meta-regression could only be performed for the syntheses that included ten or more studies.

#### Atypical Sexuality

##### Multiple Paraphilias

Five studies were synthesized to determine the association between victim age polymorphism and multiple paraphilias. A fixed-effects model revealed that individuals who are polymorphic were more likely to have two or more paraphilias compared with individuals with child victims, and the effect size was small, log *RR* = 0.28, 95% CI[0.11, 0.46], *I*^*2*^ = 2.2%. Notably, there was one outlier (Brouillette-Alarie et al., 2018) that accounted for 80.4% of the weight in the synthesis, which is likely because the study had a much larger sample size (*n* = 558) than the other four studies included in the synthesis. After removing the outlier, the effect size became negligible, log *RR* = 0.07, 95% CI[−0.34, 0.47], *I*^*2*^ = 0.0%. A random-effects model also revealed that individuals who are polymorphic were more likely to have two or more paraphilias compared with individuals with child victims, and the effect size was small, log *RR* = 0.26, 95% CI[−0.06, 0.58], *I*^*2*^ = 9.5%. Again, Brouillette-Alarie et al. (2018) was an outlier, accounting for 69.9% of the weight in the synthesis. With the outlier removed, the effect size was negligible, log *RR* = 0.07, 95% CI[−0.56, 0.69], *I*^*2*^ = 0.5%.

Compared with individuals with adult victims, those who are polymorphic were more likely to have two or more paraphilias using a fixed-effects model, and the effect size was large, log *RR* = 0.86, 95% CI[0.59, 1.13], *I*^*2*^ = 64.7%. Brouillette-Alarie et al. (2018) was an outlier, comprising 73.9% of the weight in the fixed-effects model. With the outlier removed, the effect size became small, log *RR* = 0.24, 95% CI[−0.29, 0.77], *I*^*2*^ = 29.1%. Using a random-effects model, those who are polymorphic were more likely to have two or more paraphilias than those with adult victims, and the effect size was medium, log *RR* = 0.52, 95% CI[−0.33, 1.37], *I*^*2*^ = 62.4%.

##### Sexual Preoccupation

Three studies were synthesized to determine the association between victim age polymorphism and sexual preoccupation. There was no difference in sexual preoccupation between those who are polymorphic and those with child victims with negligible effect sizes using both fixed-effects (*d* = 0.12, 95% CI[−0.07, 0.31], *I*^2^ = 0.0%) and random-effects models, *d* = 0.12, 95% CI[−0.15, 0.40], *I*^2^ = 0.0%. Similarly, there was no difference in sexual preoccupation between individuals who are polymorphic and individuals with adult victims using a fixed-effects model, and the effect size was negligible, *d* = 0.08, 95% CI[−0.14, 0.31], *I*^2^ = 79.4%. Brouillette-Alarie et al. (2018) was an outlier in the fixed-effects model, accounting for 57.9% of the weight in the synthesis. The outlier was removed, and the fixed-effects analysis revealed a small effect, *d* = 0.31, 95% CI[−0.04, 0.65], *I*^2^ = 85.5%. Using a random effects model, there was no difference in sexual preoccupation between individuals who are polymorphic and individuals who target adults, and the effect size was negligible, *d* = 0.18, 95% CI[−1.09, 1.46], *I*^2^ = 82.0%.

#### Antisociality

##### PCL-R Total Scores

Ten studies were synthesized to determine the association between psychopathy (PCL-R total score) and victim age polymorphism. Compared with individuals with only child victims, individuals who are polymorphic had higher total PCL-R scores using a fixed-effects model, and the effect size was small, *d* = 0.41, 95% CI[0.29, 0.54], *I*^2^ = 67.7%. Moderator analyses revealed that neither victim age cutoff nor information source moderated the PCL-R total effect size compared with individuals with child victims. Using a random-effects model, individuals who are polymorphic had higher total PCL-R scores compared with those with child victims, and the effect size was medium, *d* = 0.52, 95% CI[0.25, 0.79], *I*^2^ = 67.5%. Again, moderator analyses revealed neither victim age cutoff nor information sources moderated the effect size.

Using a fixed-effects model, there was no difference in PCL-R total scores between individuals who are polymorphic and those who target adults, *d* = 0.10, 95% CI[−0.04, 0.25], *I*^2^ = 80.7%. The between-level *Q* statistic indicated that information source was a significant moderator. As shown in Table 7, individuals who are polymorphic had higher PCL-R total scores compared with those with adult victims when only official records were used. When both official and self-report records were used, individuals who are polymorphic scored lower on the PCL-R compared with those with adult victims. Using a random-effects model, there was no difference in PCL-R total scores between individuals who are polymorphic and those who target adults (*d* = −0.02, 95% CI[−0.46, 0.41], *I*^2^ = 82.7%), and information sources did not moderate the effect size.

**Table 7.** Between-Level Q Subgroup Analyses for Information Sources

Variable	ISOVAP comparison	Information sources	Fixed-effects model			Random-effects model		
			Log *RR* [95% CI]	*d* [95% CI]	*Q*	Log *RR* [95% CI]	*d* [95% CI]	*Q*
Overall PCL-R scores	ISOC	Official		0.40 [0.25, 0.56]	26.05*		0.58 [0.18, 0.98]	26.05*
		Official and self-report		0.45 [0.21, 0.66]	1.76		0.43 [−0.04, 0.90]	1.76
		Between-level *Q*			0.05			0.57
	ISOA	Official		0.26 [0.08, 0.43]	24.07*		0.15 [−0.38, 0.67]	24.07*
		Official and self-report		−0.24 [−0.49, 0.02]	7.66*		−0.39 [−1.96, 1.18]	7.66*
		Between-level *Q*			9.69*			1.61
Factor 1 scores	ISOC	Official		0.30 [0.14, 0.46]	13.77*		0.33 [0.06, 0.60]	13.77*
		Official and self-report		0.34 [−0.02, 0.70]	1.08		0.34 [−2.09, 2.77]	1.08
		Between-level *Q*			0.04			6.36*
	ISOA	Official		0.21 [0.04, 0.38]	10.05		0.18 [−0.17, 0.53]	10.05
		Official and self-report		−0.43 [−0.90, 0.04]	2.51		−0.50 [−5.30, 4.38]	2.51
		Between-level *Q*			0.002			1.62
Factor 2 scores	ISOC	Official		0.44 [0.28, 0.59]	18.35*		0.52 [0.16, 0.88]	18.35*
		Official and self-report		0.48 [0.12, 0.84]	1.05		0.48 [−1.93, 2.89]	1.05
		Between-level *Q*			0.05			0.03
	ISOA	Official		−0.06 [−0.23, 0.10]	11.33*		−0.10 [−0.44, 0.24]	11.33
		Official and self-report		0.30 [0.77, 0.18]	7.39*		−0.37 [−8.79, 8.05]	7.39*
		Between-level *Q*			0.83			1.62
Sexual recidivism	ISOC	Official	0.31 [0.10, 0.52]		5.39	0.23 [−0.10, 0.55]		5.39
		Official and self-report	0.10 [−0.07, 0.28]		25.23*	0.48 [−1.01, 1.96]		25.23*
		Between-level *Q*			2.15			0.45
	ISOA	Official	0.12 [−0.06, 0.36]		10.91	0.11 [−0.41, 0.62]		10.91
		Official and self-report	0.14 [−0.06, 0.34]		12.69*	0.33 [−0.83, 1.50]		12.69*
		Between-level *Q*			0.001			0.45
Violent recidivism	ISOC	Official	0.64 [0.42, 0.86]		7.52	0.75 [0.16, 1.34]		7.52
		Official and self-report	0.36 [−0.05, 0.77]		0.70	0.36 [−0.19, 2.60]		0.70
		Between-level *Q*			1.36			1.95
	ISOA	Official	−0.15 [−0.35, 0.04]		8.83	−0.15 [−0.56, 0.25]		8.83
		Official and self-report	−0.48 [−0.88, −0.09]		0.002	−0.48 [−0.60, −0.36]		0.002
		Between-level *Q*			2.14			5.04*

*Note.* ISOC = Individuals with sexual offenses against children; ISOA = Individuals with sexual offenses against adults; ISOVAP = Individuals with sexual offenses that are victim age polymorphic; PCL-R = Psychopathy Checklist-Revised.

*p_*Q*_ < .05.

##### Factor 1 Scores

Nine studies were synthesized to examine the association between victim age polymorphism and PCL-R Factor 1 scores. Using a fixed-effects model, individuals who are polymorphic scored higher on Factor 1 compared with individuals with child victims, and the effect size was small, *d* = 0.31, 95% CI[0.16, 0.45], *I*^2^ = 46.3%. Information source did not moderate the effect size. Similarly, a random-effects model revealed that individuals who are polymorphic scored higher on Factor 1 compared with individuals with child victims, and again, the effect size was small, *d* = 0.33, 95% CI[0.12, 0.54], *I*^2^ = 46.1%. The between-level *Q* statistic indicated that information source was a significant moderator, although the overall effect sizes for both subgroups (only official records versus both official and self-report records) revealed a small association between polymorphism and Factor 1 scores compared with individuals with child victims.

There was no difference in Factor 1 scores between individuals who are polymorphic and individuals with adult victims using both fixed-effects (*d* = 0.13, 95% CI[−0.03, 0.30], *I*^2^ = 63.0%) and random-effects models, *d* = 0.05, 95% CI[−0.31, 0.41], *I*^2^ = 65.6%. Information source did not moderate the effect sizes in either of the syntheses.

##### Factor 2 Scores

Nine studies were synthesized to examine the association between victim age polymorphism and PCL-R Factor 2 scores. Compared with individuals with child victims, individuals who are polymorphic scored higher on Factor 2 using a fixed-effects model, and the effect size was small, *d* = 0.44, 95% CI[0.30, 0.58], *I*^2^ = 58.9%. Information source did not moderate the effect size. Similar results were found using a random-effects model, but this time the effect size was medium, *d* = 0.50, 95% CI[0.23, 0.77], *I*^2^ = 59.9%. Again, information source did not moderate the effect size.

There was no difference in Factor 2 scores between individuals who are polymorphic and those with adult victims using both fixed-effects (*d* = −0.09, 95% CI[−0.24, 0.07], *I*^2^ = 64.2%) and random-effects models, *d* = −0.15, 95% CI[−0.51, 0.21], *I*^2^ = 66.6%. The between-level *Q* statistic indicated that information source did not moderate the effect size in either synthesis.

#### Recidivism

##### Overall Recidivism

Across the four studies that examined overall recidivism, there was a recidivism rate of 42.2% for those who are polymorphic (*n* = 592), 37.3% for individuals with child victims (*n* = 1,221), and 49.4% for individuals with adult victims (*n* = 589). Individuals who are polymorphic were more likely than individuals with child victims to recidivate and the effect sizes were small using both fixed-effects (log *RR* = 0.19, 95% CI[0.08, 0.30], *I*^2^ = 77.9%) and random-effects models, log *RR* = 0.19, 95% CI[−0.25, 0.63], *I*^*2*^ = 79.5%.

There was no difference in overall recidivism between individuals who are polymorphic and those with adult victims with negligible effect sizes using both fixed-effects (log *RR* = −0.13, 95% CI[−0.24, −0.02], *I*^*2*^ = 24.8%) and random-effects models, log *RR* = −0.12, 95% CI[−0.34, 0.10], *I*^*2*^ = 21.3%.

##### Sexual Recidivism

Across the ten studies that examined sexual recidivism, there was a sexual recidivism rate of 14.6% for individuals who are polymorphic (*n* = 1,325), 13.4% for individuals with child victims (*n* = 3,649), and 14.6% for individuals with adult victims (*n* = 2,055). Using a fixed-effects model, individuals who are polymorphic were more likely to recidivate sexually than individuals with child victims, and the effect size was small, log *RR* = 0.19, 95% CI[0.06, 0.32], *I*^*2*^ = 72.6%. Moderator analyses revealed that victim age cutoff, follow up time, and information source did not moderate the effect size. Similar results were found using a random-effects model, log *RR* = 0.28, 95% CI[−0.01, 0.57], *I*^*2*^ = 67.7%. Moderator analyses again revealed that victim age cutoff, follow up time, and information source did not moderate the effect size.

There was no difference in sexual recidivism between individuals who are polymorphic and individuals with adult victims using both fixed-effects (log *RR* = 0.11, 95% CI[−0.03, 0.24], *I*^*2*^ = 66.8%) and random-effects models, log *RR* = 0.13, 95% CI[−0.20, 0.47], *I*^*2*^ = 71.7%. Moderator analyses revealed that victim age cutoff, follow up time, and information source did not moderate the effect size for either of the syntheses.

##### Violent Recidivism

Across the seven studies that examined violent recidivism, there was a nonsexual violent recidivism rate of 22.6% for individuals who are polymorphic (*n* = 727), 12.5% for individuals with child victims (*n* = 1,882), and 29.9% for individuals with adult victims (*n* = 1,030). Individuals who are polymorphic were more likely to recidivate violently than individuals with child victims using a fixed-effects model, and the effect size was medium, log *RR* = 0.58, 95% CI[0.39, 0.77], *I*^*2*^ = 37.4%. Information source did not moderate the effect size. There was one notable outlier (Howard et al., 2014) in the synthesis, which comprised 56.6% of the weight in the fixed-effects model, likely due to the very large sample size in this study (*n* = 1,586). After removing the outlier, the effect size remained medium, log *RR* = 0.63, 95% CI[0.34, 0.92], *I*^*2*^ = 46.6%. Using a random-effects model, individuals who are polymorphic were more likely to recidivate violently than individuals with child victims, and the effect size was medium, log *RR* = 0.61, 95% CI[0.23, 0.99], *I*^*2*^ = 32.2%. Again, information source did not moderate the effect size, and Howard et al. (2014) was an outlier in the synthesis. After removing the outlier, the effect size using the random-effects model remained medium, log *RR* = 0.66, 95% CI[0.12, 1.20], *I*^*2*^ = 46.5%.

Compared with individuals with adult victims, those who are polymorphic were less likely to recidivate violently using a fixed-effects model, and the effect size was small, log *RR* = −0.22, 95% CI[−0.39, −0.05], *I*^*2*^ = 45.3%. Information source did not moderate the effect size. Howard et al. (2014) was identified as an outlier in the synthesis, accounting for 54.0% of the weight. Similar results were found after removing the outlier, log *RR* = −0.28, 95% CI[−0.54, −0.03], *I*^*2*^ = 52.4%. Using a random-effects model, individuals who are polymorphic were less likely to recidivate violently than those who target adults, and the effect size was small, log *RR* = −0.22, 95% CI[−0.51, 0.07], *I*^*2*^ = 0.0%. The between-level *Q* statistic indicated that information source was a significant moderator. As shown in Table 7, the overall effect size of studies using only official records was negligible. For studies using both official and self-report records, individuals who are polymorphic were less likely to recidivate violently than those with adult victims, and the effect size was medium. Again, Howard et al. (2014) was an outlier, accounting for 54.0% of the weight in the random-effects synthesis. After removing the outlier, results again indicated that individuals who are polymorphic were less likely to recidivate violently than individuals with adult victims, and the effect size was small, log *RR* = −0.28, 95% CI[−0.77, 0.21], *I*^*2*^ = 5.1%.^2^

## Discussion

In summary, fixed-effects syntheses revealed that individuals who are polymorphic were more likely to have multiple paraphilia diagnoses compared with individuals with child victims or adult victims. They also scored higher on the PCL-R (total, Factor 1, and Factor 2) compared with individuals with child victims, but not those with adult victims. Lastly, individuals who are polymorphic had higher overall, sexual and nonsexual violent recidivism compared with individuals with child victims, and lower nonsexual violent recidivism compared with individuals with adult victims. Therefore, individuals who are polymorphic demonstrate more similarities to individuals with adult victims than individuals with child victims in terms of antisociality and sexual recidivism, but may be differentiated from both non-polymorphic groups based on a greater number of paraphilias.

A random-effects model is generally preferred in meta-analysis when notable heterogeneity is present between studies; however, the number of studies included in the present review was small enough to raise concerns about the stability of the effect sizes found here. Therefore, this discussion places emphasis on the results of the fixed-effects analyses. While the effect sizes found in the present study generally followed the same trend across fixed- and random-effects models, it should be noted that several of the effect sizes differed in magnitude. For example, the large effect size for the association between polymorphism and multiple paraphilias compared with those with adult victims became a medium effect size using a random-effects model.

Further, it is noteworthy that heterogeneity was high across several analyses. Although the plan was to examine the impact of various methodological differences on the findings, we were only able to examine moderators for a limited number of analyses. Overall, victim age cutoff did not appear to moderate the findings in the three syntheses for which it could be examined (i.e., PCL-R total scores compared with individuals with child victims, sexual recidivism compared with both non-polymorphic groups). Information source moderated the effect size for just three out of ten syntheses for which it could be examined; however, there was no consistent pattern across syntheses, nor was there consistency between fixed- and random-effects models, that could explain the findings. Given these disparate findings, it is unclear what effect, if any, moderators may have on the present analyses.

### Atypical Sexuality and Sexual Recidivism

While the atypical sexuality results were based only on a few studies, the effect sizes that emerged in this domain were notable. The association between victim age polymorphism and multiple paraphilias provides some preliminary support for the sexualization pathway of offending among individuals who are polymorphic. The sexualization hypothesis (encompassing a wide range of constructs such as paraphilias and sexual preoccupation) states that the inability to control sexual behaviour and/or urges may lead to sexual offending against multiple types of victims (Knight & Sims‐Knight, 2003; Lussier et al., 2007). Although polymorphism was associated with multiple paraphilias in the present review, it is important to note that there was no association between polymorphism and sexual preoccupation based on a small number of studies.

In line with previous research (e.g., Hanson & Morton-Bourgon, 2005), the effect sizes in the atypical sexuality domain may have contributed to the association between victim age polymorphism and sexual recidivism compared with individuals with child victims (no differences were found between individuals who are polymorphic and individuals with adult victims). Across studies, individuals with child victims demonstrated a lower sexual recidivism rate than both individuals with adult victims and individuals who are polymorphic. It is possible that this finding may be a function of combining individuals with extrafamilial and intrafamilial child victims into one group because individuals with extrafamilial child victims are known to have a higher sexual recidivism rate than individuals with intrafamilial child victims (19.5% and 8.4%, respectively; Hanson, 2001). On the other hand, it would also make sense that those who are polymorphic would have higher sexual recidivism rates given their elevated scores in the atypical sexuality domain, as well as their greater potential victim pool (Stephens et al., 2017).

Although various methodological moderators of sexual recidivism were examined and not statistically significant, we were unable to examine the effect of excluding individuals with sexual offenses with a single victim on recidivism findings. Thus, the present study does not exclude the possibility that the association between sexual recidivism and victim age polymorphism is explained by having multiple victims, which is relevant because previous research found this association disappeared when controlling for the number of victims (Stephens et al., 2016). Readers should interpret these results cautiously and should not take the higher rate of recidivism to mean that victim age polymorphism might add incremental validity to pre-established risk tools.

### Psychopathy and Violent Recidivism

The association between psychopathy and victim age polymorphism was much more robust given the number of studies included in the analysis. Based on these results, individuals who are polymorphic demonstrate more similarities to individuals with adult victims on the PCL-R than individuals with child victims, suggesting that a history of sexual offending against adults is associated with elevated psychopathy scores. Importantly, elevated PCL-R scores among those who are polymorphic could translate into higher rates of violent recidivism. The findings of the present meta-analysis support this hypothesis given that individuals who are polymorphic had both higher PCL-R scores and higher violent recidivism compared with individuals with child victims; however, the hypothesis was not supported when compared with individuals with adult victims, as those who are polymorphic had similar PCL-R scores but lower violent recidivism. Nonetheless, these findings suggest that a possible driver of polymorphism is likely related to antisociality and associated deficits.

It is interesting to note that there is a relationship between psychopathy and sexual preoccupation in both community (e.g., Dyer & Olver, 2016; Lee & Forbey, 2010) and justice-involved samples (e.g., Harris et al., 2007). Issues with self-regulation, novelty-seeking, and impulsivity have been theorized to drive the association between sexual preoccupation and psychopathy (Kastner & Sellbom, 2012; Meloy, 2002). Novelty-seeking is thought to contribute to increased likelihood of individuals seeking different types of victims, which may lead to victim age polymorphism (Porter et al., 2000). Thus, it is suspected that novelty-seeking could be an important factor to examine among those who are polymorphic. Although sexual preoccupation did not differ across groups in the present study, this finding is based on just three studies and should be explored further. Overall, it is important to examine the relationship between self-regulation and victim age polymorphism given the implications self-regulation issues would have for clinicians tasked with providing psychological treatment to individuals who are polymorphic.

### Implications

The first major takeaway of the present study is that individuals who are polymorphic demonstrated more similarities to individuals with adult victims than individuals with child victims. Compared with individuals with child victims, those who are polymorphic demonstrated elevated scores across all variables with the exception of sexual preoccupation. These findings could have implications for the identification of individuals with sexual offenses who may exhibit victim age polymorphism in future offenses, which is an important consideration in supervision planning and child protection. For example, consider a question from a child protection worker who wonders if someone who has offended against a young adult should have unsupervised access to their eight-year-old child. As another example, a parole officer may be working with someone who has exclusively offended against children and wonders if romantic relationships with adults should be monitored. Research on victim age polymorphism can provide some insight into the answers to these questions. The results of the present review might indicate that individuals with non-polymorphic sexual offenses who have elevated rates of psychopathy and multiple paraphilias may be at risk of targeting other age groups in future offenses. Of course, an important caveat is that there has been no research to date that has prospectively followed non-polymorphic individuals to examine what factors predict polymorphism in future sexual offences. Thus, caution is needed when relying on retrospective research to inform release planning and risk monitoring.

The second major takeaway pertains to implications for the clinical assessment and treatment of individuals who are polymorphic. Overall, it appears that victim age polymorphism is associated with a general antisocial predisposition, which in turn is linked to some aspects of atypical sexuality. Thus, individuals who are polymorphic may have greater difficulties in the risk-related domains of antisociality and atypical sexuality, which could put them at a heightened risk of sexual recidivism compared with those with elevations in only one of those domains (Hawes et al., 2013). Relatedly, the findings suggest that individuals who are polymorphic may also have a wide breadth of more generalized criminogenic needs to address during clinical assessment and treatment. Clinicians should carefully consider whether individuals who are polymorphic require a more generalized approach in treatment to reduce risk of sexual and non-sexual recidivism, compared with a more sex-offense specific treatment approach.

### Limitations

The present study should be interpreted with several limitations in mind. Most importantly, the study was limited by the lack of available data in multiple ways. First, the small number of studies included in the syntheses means effect sizes were unstable, and fixed-effects models were more appropriate to interpret than random-effects models even though it is expected that effect sizes vary based on more than just sampling error. Many syntheses had high heterogeneity and some outliers were present, particularly in the few studies that examined the relationship between atypical sexuality and victim age polymorphism. As a result, the findings reported in the atypical sexuality domain and the conclusions drawn about this construct in individuals who are polymorphic should be interpreted with caution until additional research is conducted. Second, many studies (*n* = 18) were excluded because corresponding authors didn’t respond (*n* = 15) or were unable to share data due to restricted access (*n* = 3). While the exclusion of a high number of studies is concerning, low response rates from corresponding authors are a known issue in meta-analyses (Field & Gillett, 2010) and meta-analyses focused on sexual offending have reported response rates similar to the present study (e.g., 48% response rate in Seto & Lalumière, 2010). Third, we relied heavily on published studies. Additional methods of obtaining unpublished studies may have improved retrieval, although the procedure followed in the present study was typical for psychological research (Field & Gillett, 2010). Given the small number of studies included in some of the present analyses, even a few unpublished effect sizes could alter these findings, which represents a significant limitation.

Another limitation is that key methodological characteristics of the samples impacted our ability to perform the proposed analyses. Many continuous and categorical moderators could not be examined because there was little variation in study methodology (e.g., all but one study used child and adult victim age categories). Even in circumstances where there was sufficient variation in methodology to include a continuous moderator (e.g., victim age cutoffs), only the impact on total PCL-R scores and sexual recidivism could be examined because these met the 10-study threshold needed to perform meta-regression (Borenstein et al., 2009). Scurich and Gongola (2021) demonstrated that several methodological characteristics impacted the prevalence of victim age polymorphism. Thus, although many of these moderators could not be examined in the present study, it is expected that methodological moderators could explain some of the diversity in research on risk-related domains and recidivism.

Finally, the recidivism data reported in the studies often did not account for variations in follow up time, despite differing follow up periods in several of the studies. It is important to consider the influence of time given that cumulative recidivism rates increase over time (Durose et al., 2014). Studies included in the present review usually reported recidivism rates and the average follow up time of the entire sample, but did not account for follow up time in the associations between polymorphism and recidivism via analyses such as Cox regression. The potential influence of follow up time is especially concerning given empirical evidence that individuals with child victims generally have longer detection times than individuals with adult victims (Lussier et al., 2011); that is, studies with shorter follow up time may not accurately capture the recidivism rates of individuals with child victims because the offenses are not discovered until many years later. Therefore, the possibility that follow up time influenced recidivism findings in the present study cannot be entirely ruled out.

### Future Research Directions

The present study identifies several avenues for future research to increase our understanding of victim age polymorphism. First, the literature on victim age polymorphism and atypical sexuality is lacking, even though preliminary evidence suggests that certain manifestations of atypical sexuality are important contributors to victim age polymorphism. Future research should examine the relationship between victim age polymorphism and other measures of atypical sexuality, particularly those associated with psychopathy (e.g., sexual preoccupation, sensation-seeking). Additionally, the results of Stephens et al. (2023) provide some evidence that victim age polymorphism is associated with indiscriminate arousal, albeit the size of the effect was small. There is a need to replicate and explore the relationship between victim age polymorphism and indiscriminate arousal in future research given the implications for clinical assessment. It is possible that indiscriminate arousal in individuals with sexual offenses could enable the identification of those who are at risk of targeting different age groups in future offenses.

A related area of inquiry would be to conduct a prospective study examining the role of various risk factors in determining whether an individual with a sexual offense becomes polymorphic. The present study and extant literature provide several candidate risk factors, but to our knowledge, there has not been any examination of whether these factors predict who will become polymorphic. Research like this could have significant implications for a wide variety of agencies. For example, child protection workers often have questions about the risk an individual client poses to a group that differs from their previous victim(s). Further prospective research could be helpful in providing answers to these questions.

Lastly, the literature on victim age polymorphism and psychopathy is more fulsome, although an item-level analysis of the PCL-R may elucidate distinctions between individuals with adult victims and those who are polymorphic. Specifically, proneness to boredom and other items related to thrill-seeking and sensation-seeking may explain the motivation to target multiple age groups across sexual offenses (Porter et al., 2000). Thus, individuals who are polymorphic may score higher on these PCL-R items and related measures (e.g., sensation-seeking, impulsivity) than individuals with adult victims.

## Supplemental Material

Supplemental Material - A Meta-Analysis of Atypical Sexuality, Psychopathy, and Recidivism Associated With Victim Age Polymorphism Supplemental Material for A Meta-Analysis of Atypical Sexuality, Psychopathy, and Recidivism Associated With Victim Age Polymorphism by Samantha K. Williams, Desiree L. Elchuk, Skye Stephens in Sexual Abuse.

## Notes

1. Indiscriminate sexual arousal data were sought; however, only two studies meeting the inclusion criteria were found. Michaud and Proulx (2009) and Stephens et al. (2023) used different methods and stimuli and therefore could not be synthesized. Michaud and Proulx (2009) detected an indiscriminate arousal profile among individuals who are polymorphic, although the sample size of the polymorphic group was quite small (*N* = 29). Stephens and Colleagues (2023) found a small association between victim age polymorphism and non-exclusive age interest. Taken together, these findings suggest indiscriminate sexual arousal may be an important factor to consider in the assessment of those who are polymorphic, although further research is needed.

2. Parent et al. (2011) examined sexual and violent recidivism at 5-, 10-, and 15-year follow up periods. The 10-year period was included in the analyses because it was closest to the average follow up period across all recidivism studies (*M* = 10.11 years); however, Parent et al. (2011) was weighted heavily in the analyses. To account for possible changes in results, analyses were run with the 5- and 15-year follow up periods to check for major differences using fixed-effects models. Effect sizes remained the same or similar (all within log *RR* ± 0.10 of effect sizes found using 10-year follow up period) *with the following exceptions:* Sexual recidivism compared with individuals with adult victims at the 5-year follow up (log *RR* = 0.01, 95% CI[−0.14, 0.15], *I*^2^ = 56.1%); Violent recidivism compared with individuals with adult victims at the 5-year follow up (log *RR* = −0.12, 95% CI[−0.30, 0.06], *I*^2^ = 74.9%).
