# Supplementary material for: A Meta-Analysis of Atypical Sexuality, Psychopathy, and Recidivism Associated With Victim Age Polymorphism
Source: Sex Abuse. 2026 Jan 24;38(3):273–312. doi: 10.1177/10790632261415817 (PMC12916886; doi:10.1177/10790632261415817)
Supplement: Supplemental Material - A Meta-Analysis of Atypical Sexuality, Psychopathy, and Recidivism Associated With Victim Age Polymorphism [file sj-pdf-1-sax-10.1177_10790632261415817.pdf]

Supplemental Material

Figure 1

Forest Plot of Multiple Paraphilias Effect Sizes

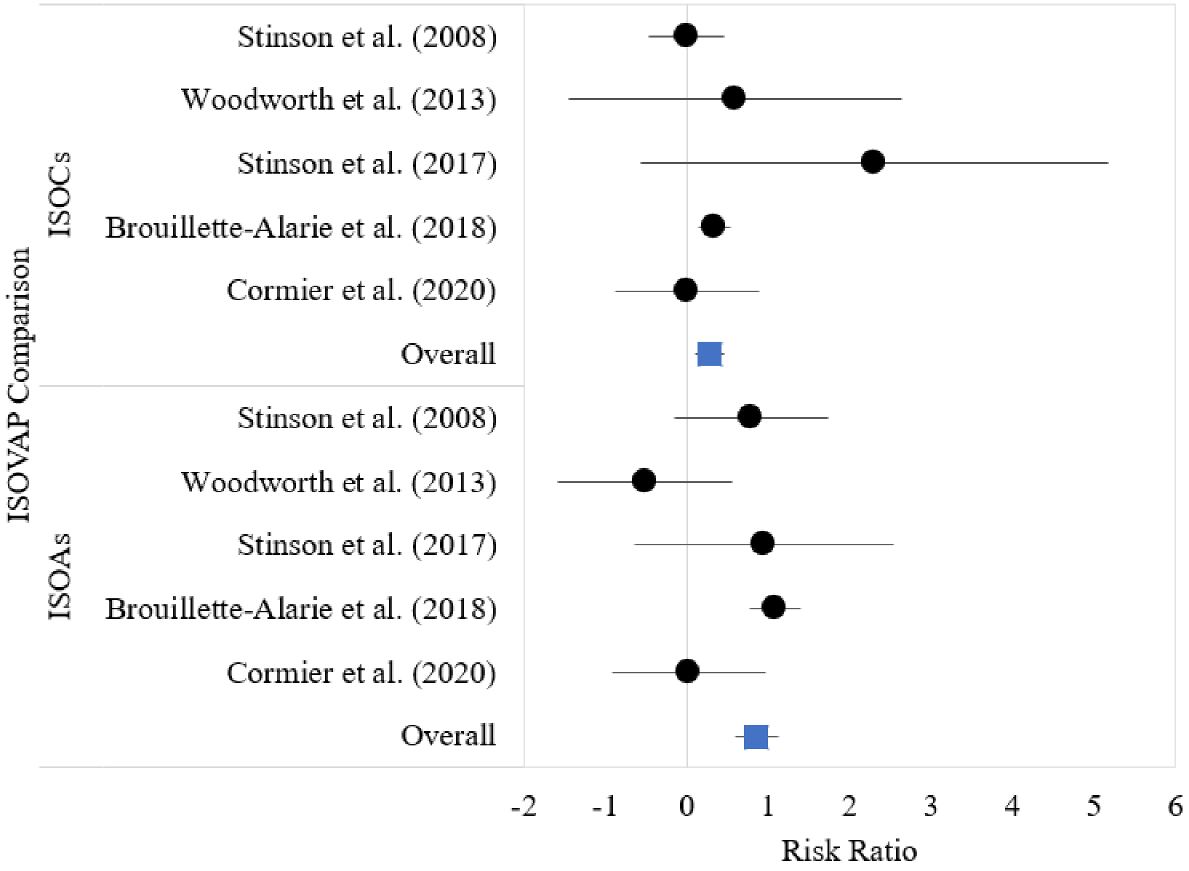

Note. k = 5. Overall effect size reports results of fixed-effects model. ISOC = Individuals with sexual offences against children; ISOA = Individuals with sexual offences against adults; ISOVAP = Individuals with sexual offences that are victim age polymorphic. Positive Risk Ratios (*RR*) indicate increased likelihood of multiple paraphilias in the ISOVAP group. Negative *RR*s indicate decreased likelihood of multiple paraphilias in the ISOVAP group.

Figure 2

Forest Plot of Sexual Preoccupation Effect Sizes

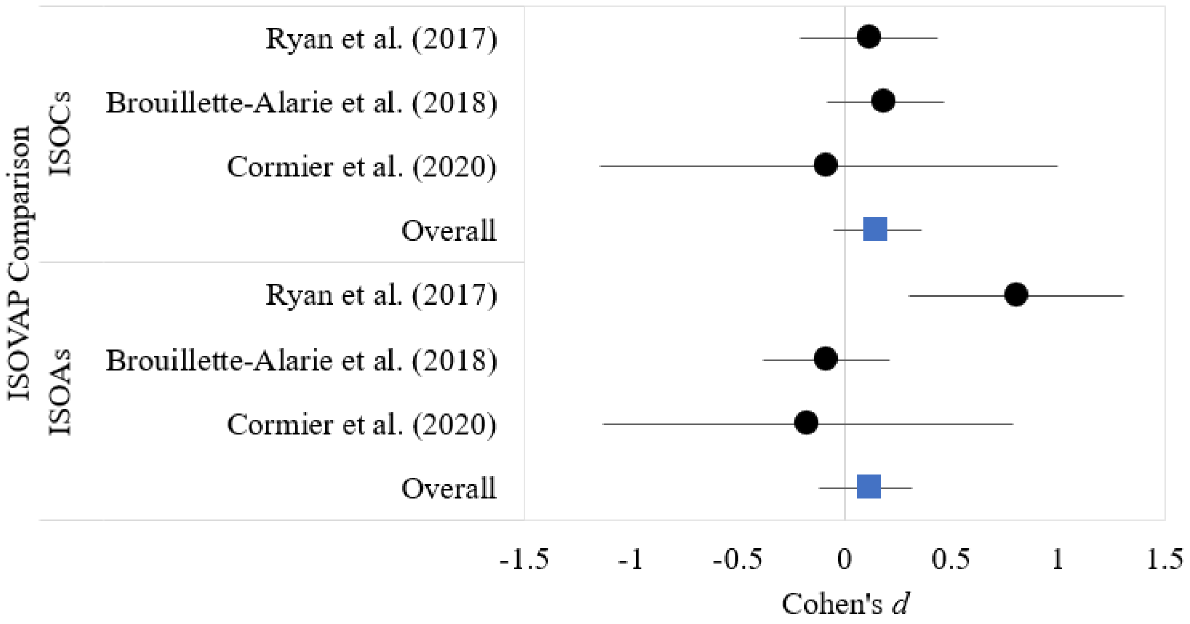

Note. k = 3. Overall effect size reports results of fixed-effects model. ISOC = Individuals with sexual offences against children; ISOA = Individuals with sexual offences against adults; ISOVAP = Individuals with sexual offences that are victim age polymorphic. Positive Cohen's *d* indicates increased association between sexual preoccupation and victim age polymorphism. Negative Cohen's *d* indicates decreased association between sexual preoccupation and victim age polymorphism.

Figure 3

Forest Plot of Total PCL-R Score Effect Sizes

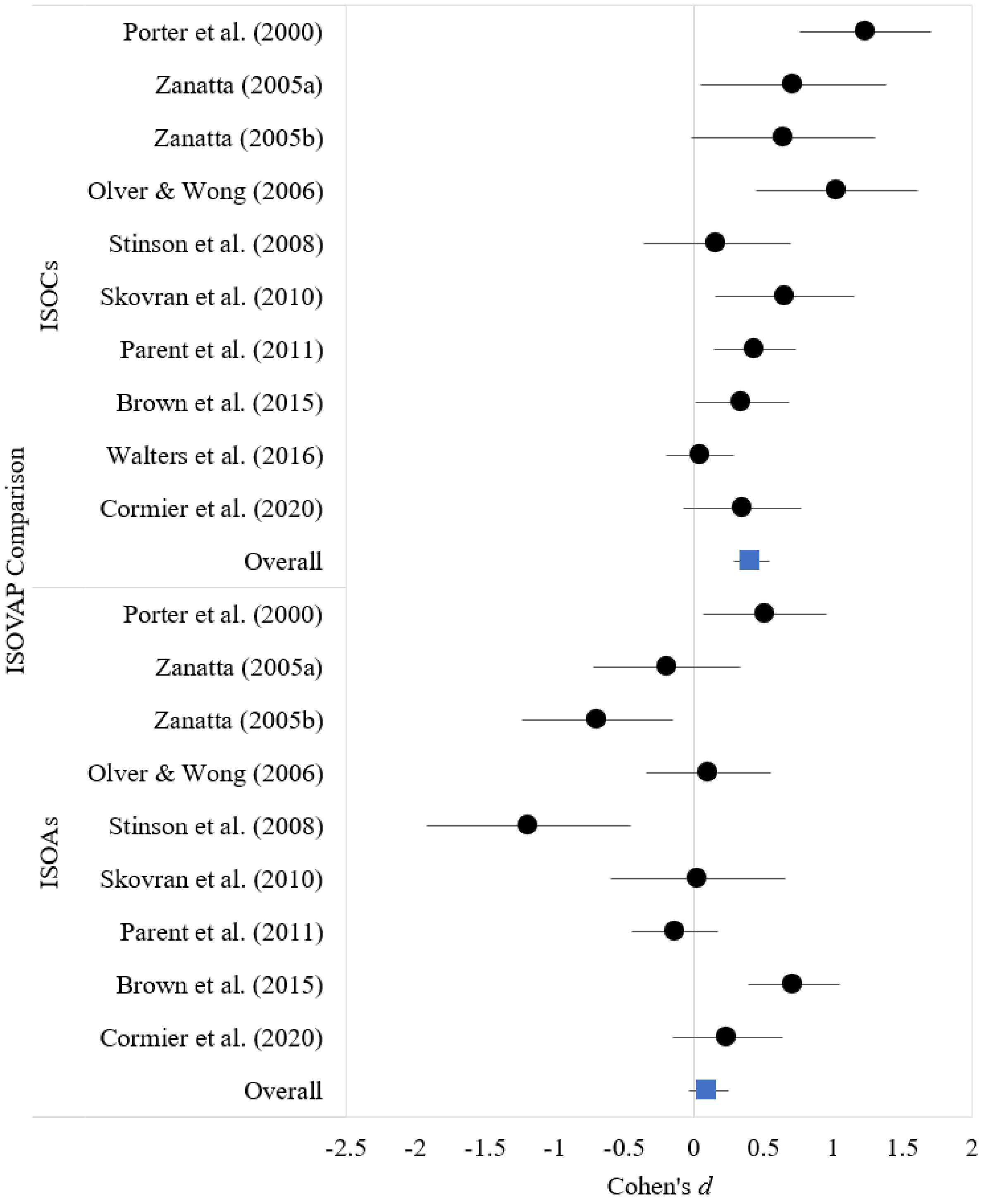

Note. k = 10. Overall effect size reports results of fixed-effects model. ISOC = Individuals with sexual offences against children; ISOA = Individuals with sexual offences against adults; ISOVAP = Individuals with sexual offences that are victim age polymorphic. Positive Cohen's *d* indicates a positive relationship between overall PCL-R scores and victim age polymorphism. Negative Cohen's *d* indicates negative relationship between overall PCL-R scores and victim age polymorphism.

Figure 4

Forest Plot of Factor 1 PCL-R Effect Sizes

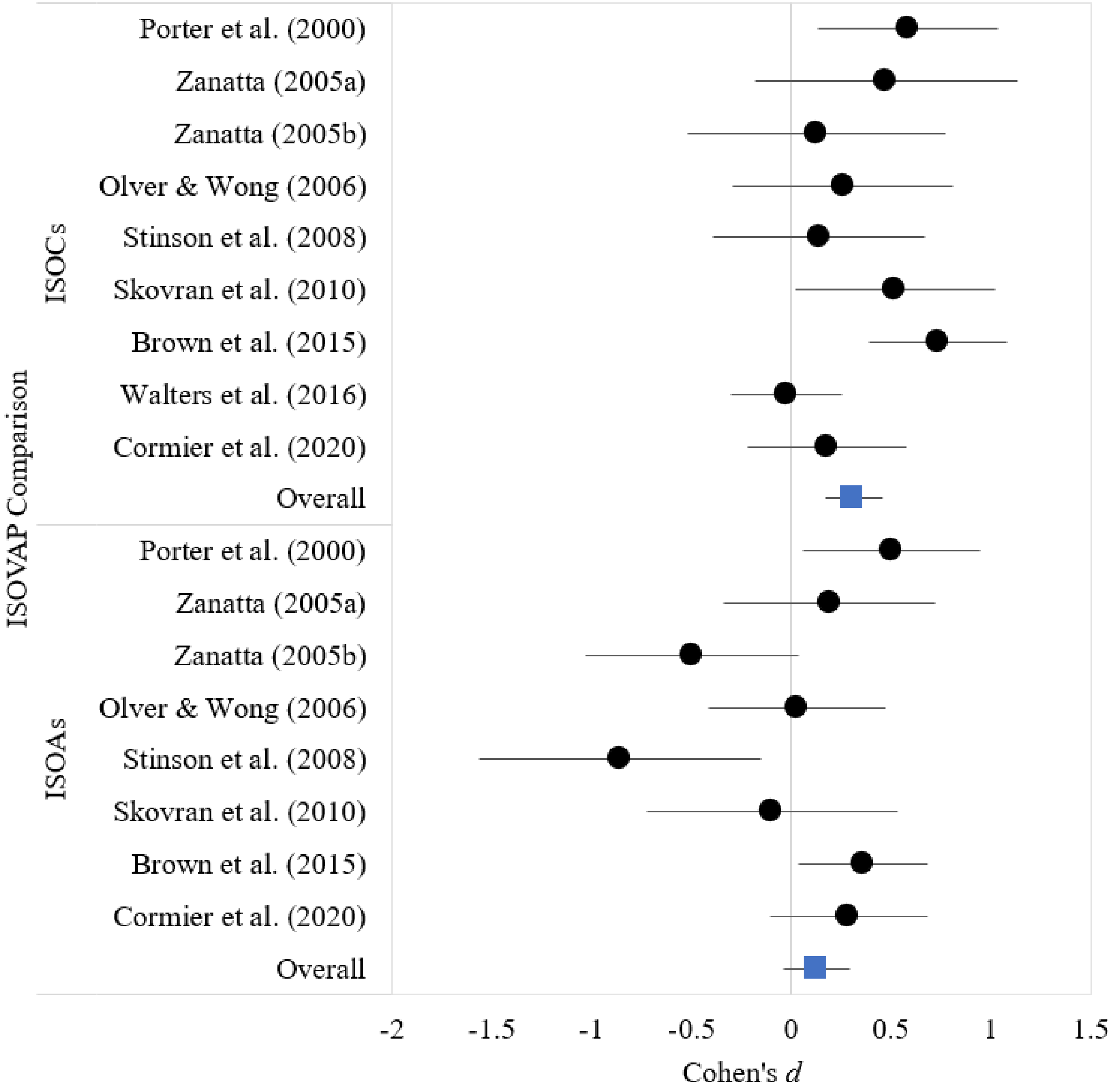

Note. k = 9*.* Overall effect size reports results of fixed-effects model. ISOC = Individuals with sexual offences against children; ISOA = Individuals with sexual offences against adults; ISOVAP = Individuals with sexual offences that are victim age polymorphic. ISOVAPs were used as the comparison group; control group is indicated on vertical axis. Positive Cohen's *d*

indicates positive relationship between Factor 1 scores and victim age polymorphism. Negative Cohen's *d* indicates negative relationship between Factor 1 scores and victim age polymorphism.

Figure 5

Forest Plot of Factor 2 PCL-R Effect Sizes

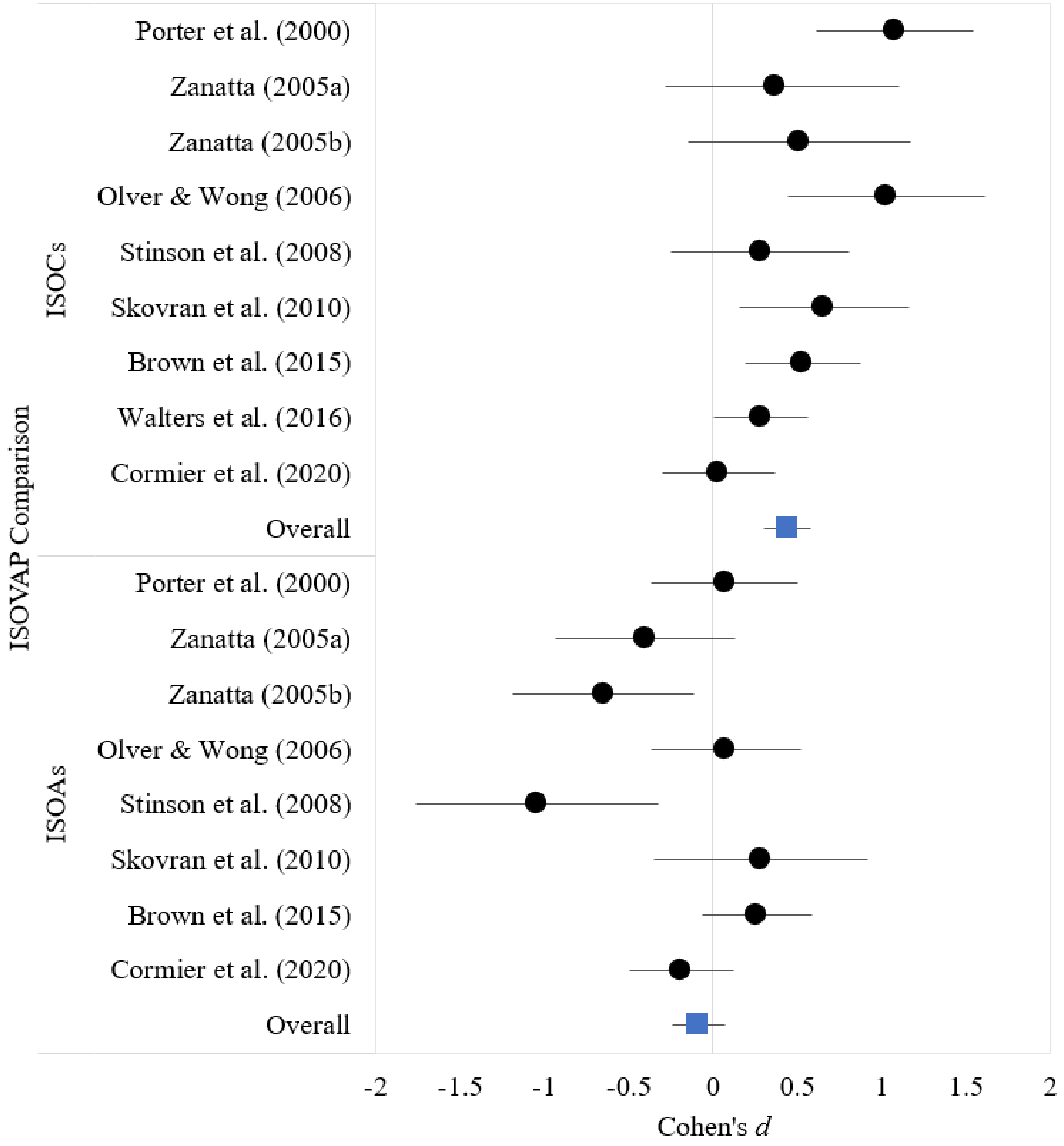

Note. k = 9. Overall effect size reports results of fixed-effects model. ISOC = Individuals with sexual offences against children; ISOA = Individuals with sexual offences against adults; ISOVAP = Individuals with sexual offences that are victim age polymorphic. Positive Cohen's *d*

indicates positive relationship between Factor 2 scores and victim age polymorphism. Negative Cohen's *d* indicates negative relationship between Factor 2 scores and victim age polymorphism.

Figure 6

Forest Plot of Overall Recidivism Effect Sizes

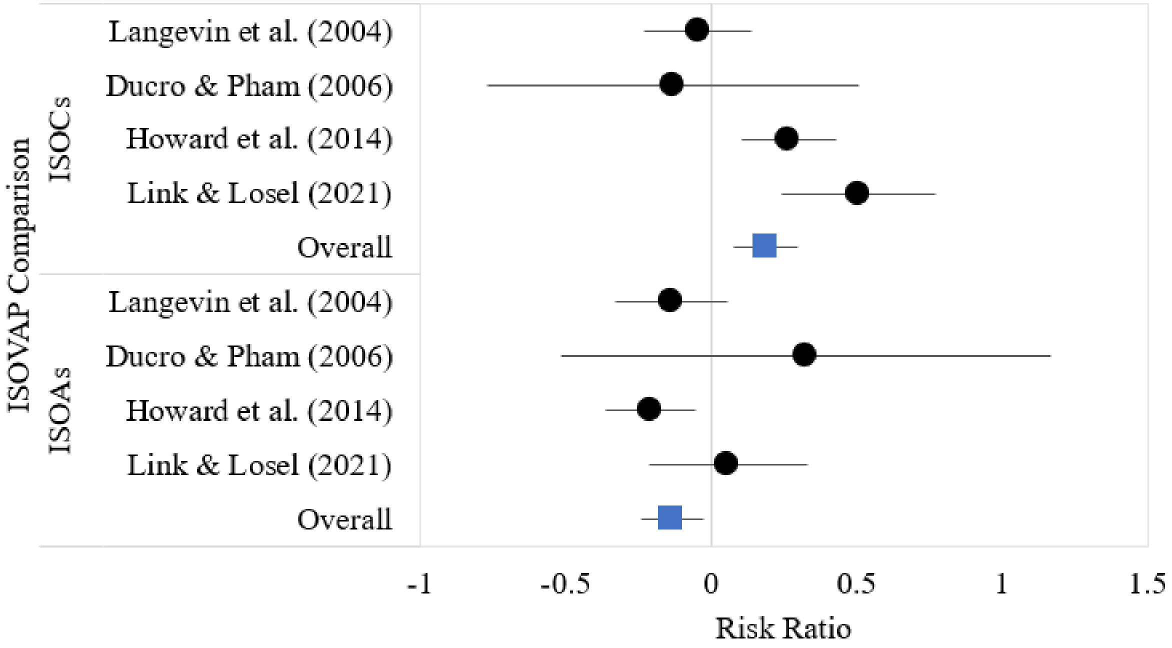

Note. k = 4. Overall effect size reports results of fixed-effects model. ISOC = Individuals with sexual offences against children; ISOA = Individuals with sexual offences against adults; ISOVAP = Individuals with sexual offences that are victim age polymorphic. Positive Risk Ratios (*RR*) indicate increased likelihood of overall recidivism in the ISOVAP group. Negative *RR*s indicate decreased likelihood of overall recidivism in the ISOVAP group.

Figure 7

Forest Plot of Sexual Recidivism Effect Sizes

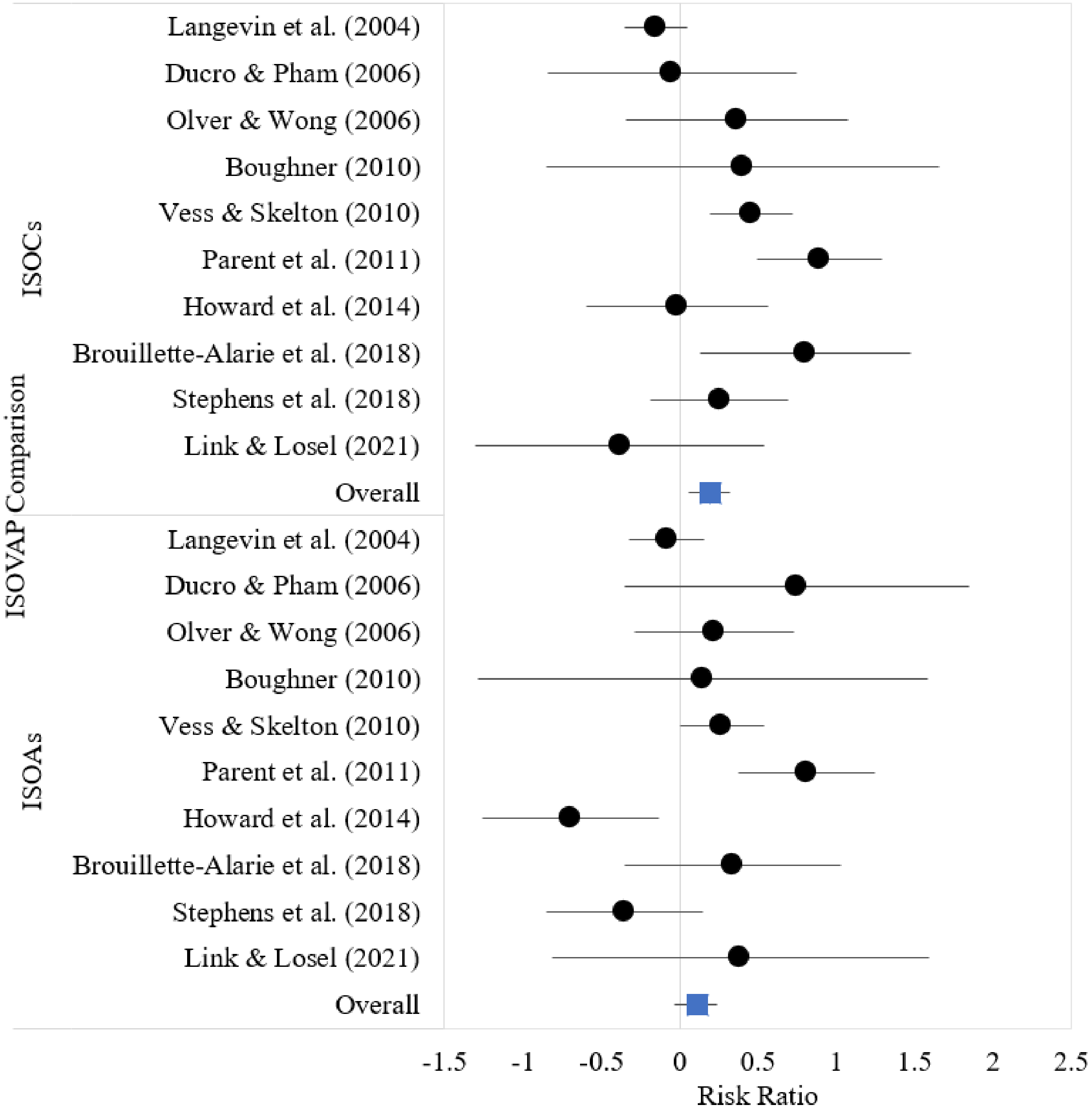

Note. k = 10. Overall effect size reports results of fixed-effects model. ISOC = Individuals with sexual offences against children; ISOA = Individuals with sexual offences against adults; ISOVAP = Individuals with sexual offences that are victim age polymorphic. Positive Risk

Ratios (*RR*) indicate increased likelihood of sexual recidivism in the ISOVAP group. Negative *RR*s indicate decreased likelihood of sexual recidivism in the ISOVAP group.

Figure 8

Forest Plot of Violent Recidivism Effect Sizes

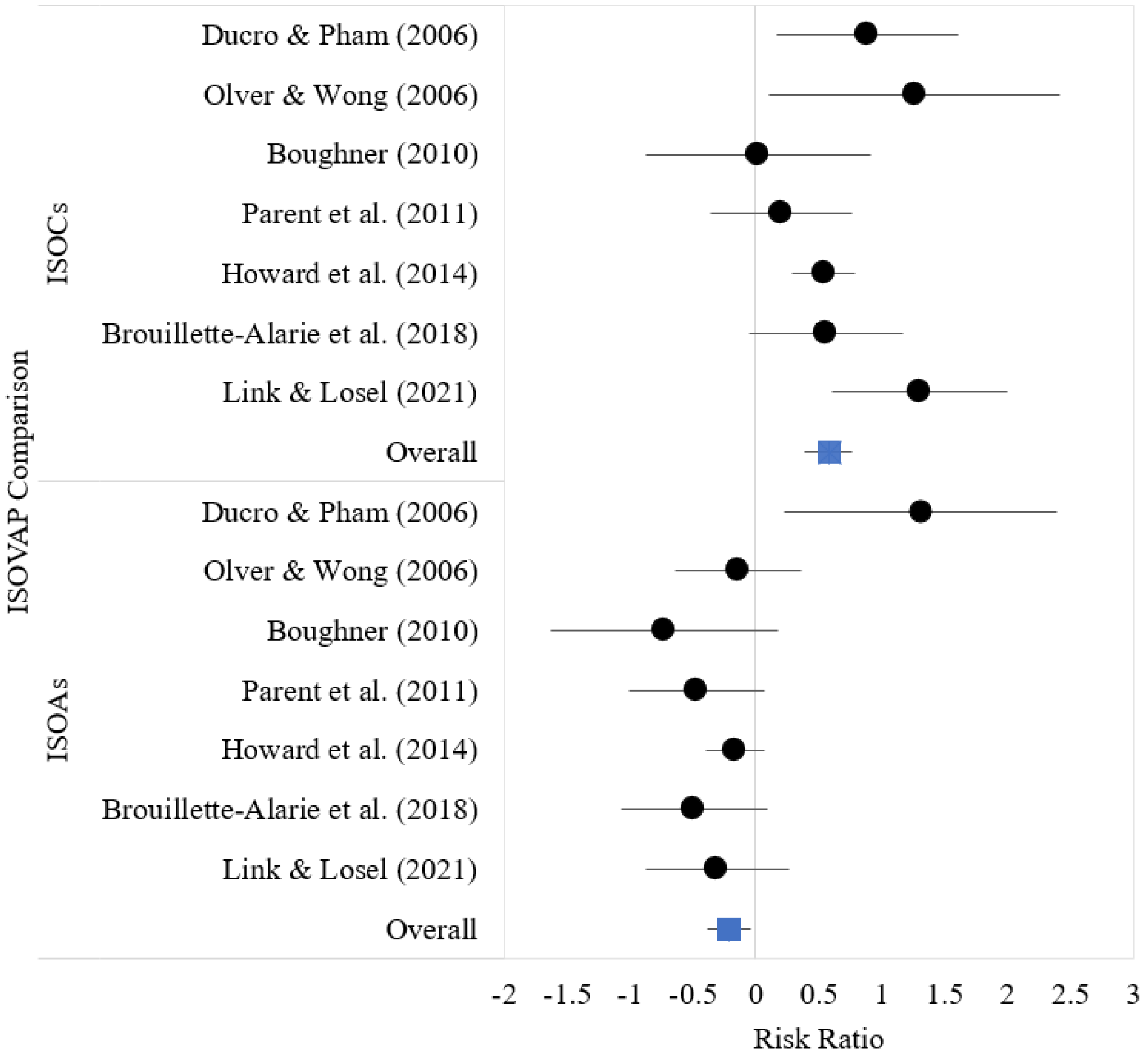

Note. k = 7. Overall effect size reports results of fixed-effects model. ISOC = Individuals with sexual offences against children; ISOA = Individuals with sexual offences against adults; ISOVAP = Individuals with sexual offences that are victim age polymorphic. Positive Risk Ratios (*RR*) indicate increased likelihood of nonsexual violent recidivism in the ISOVAP group. Negative *RR*s indicate decreased likelihood of nonsexual violent recidivism in the ISOVAP

group. ISOVAPs were significantly more likely to recidivate violently compared to ISOCs and significantly less likely to recidivate violently than ISOAs.
